# Financial risk protection from vaccines in 52 Gavi-eligible low- and middle-income countries: A modeling study

**DOI:** 10.1371/journal.pmed.1004764

**Published:** 2025-11-04

**Authors:** Boshen Jiao, Ryoko Sato, Joshua Mak, Bryan Patenaude, Margaret de Villiers, Aniruddha Deshpande, Ivane Gamkrelidze, Katy A. M. Gaythorpe, Timothy B. Hallett, Mark Jit, Xiang Li, Benjamin Lopman, Shevanthi Nayagam, Devin Razavi-Shearer, Yvonne Tam, Kim H. Woodruff, Daniel Hogan, Tewodaj Mengistu, Stéphane Verguet

**Affiliations:** 1 Leonard D. Schaeffer Center for Health Policy and Economics, University of Southern California, Los Angeles, California, United States of America; 2 Department of Pharmaceutical and Health Economics, Alfred E. Mann School of Pharmacy and Pharmaceutical Sciences, University of Southern California, Los Angeles, California, United States of America; 3 Department of Global Health and Population, Harvard T.H. Chan School of Public Health, Boston, Massachusetts, United States of America; 4 Department of International Health, Johns Hopkins University Bloomberg School of Public Health, Baltimore, Maryland, United States of America; 5 MRC Centre for Global Infectious Disease Analysis, School of Public Health, Imperial College London, London, United Kingdom; 6 Department of Epidemiology, Rollins School of Public Health, Emory University, Atlanta, Georgia, United States of America; 7 CDA Foundation, Lafayette, Colorado, United States of America; 8 Department of Infectious Disease Epidemiology and Dynamics, London School of Hygiene and Tropical Medicine, London, United Kingdom; 9 Department of Global and Environmental Health, School of Global Public Health, New York University, New York, United States of America; 10 Department of Metabolism, Digestion and Reproduction, Imperial College London, London, United Kingdom; 11 Gavi, the Vaccine Alliance, Geneva, Switzerland; Universitair Medisch Centrum UtrechtNETHERLANDS, KINGDOM OF THE

## Abstract

**Background:**

Poverty alleviation is a major global development goal. Vaccines have the potential to provide financial risk protection (FRP) by preventing illnesses and associated healthcare costs. We estimate the lifetime FRP benefits generated by major vaccines among individuals vaccinated between 2000 and 2030 in low- and middle-income countries (LMICs).

**Methods and findings:**

We developed a microsimulation model to quantify the number of cases of catastrophic health expenditure (CHE) averted by a range of vaccines in 52 Gavi-eligible countries, stratified by wealth quintile. Vaccines protecting against five pathogens were considered, i.e., hepatitis B (routine and birth dose vaccine), *Haemophilus influenzae* type B, rotavirus, measles (routine and supplementary campaign vaccine), and *Streptococcus pneumoniae*. Model inputs were obtained from secondary data sources, including infection reduction rates under various immunization coverage scenarios, out-of-pocket health expenditures, transportation costs, wage losses, and healthcare utilization associated with disease treatment and consumption expenditures. CHE cases were defined as exceeding 10% of annual consumption, with sensitivity analyses conducted using thresholds of 25% and 40%, as well as impoverishing health expenditures were estimated. All vaccines, singly and collectively, showed a large impact on FRP and could avert ~200 million CHE cases across 52 Gavi-eligible countries from 2000 to 2030. Importantly, about half of all CHE cases were prevented among the poorest quintiles. When evaluated at a 10% threshold for CHE, the first dose of measles vaccine stood out in averting around 1,400 CHE cases per 10,000 vaccinated individuals in the poorest quintile, that is a total of 44 million CHE cases averted. A key limitation is the assumption of uniform disease risks in the absence of vaccination across quintiles, which may underestimate benefits for poorer groups.

**Conclusions:**

Vaccines can provide substantial FRP benefits, particularly among the most disadvantaged populations. Sustained investments to ensure vulnerable populations receive vaccinations in LMICs can therefore not only improve health outcomes but also contribute to poverty reduction.

## Author summary

**Why was this study done?:**

- Financial risk protection (FRP) is a core element of universal health coverage, yet the FRP benefits of vaccines remain underexplored compared to their well-established health benefits.
- Out-of-pocket health expenditures are a major driver of poverty, and preventive strategies like vaccination could protect households, especially the poorest.
- More evidence is needed on how vaccines contribute to FRP across different socioeconomic groups in low- and middle-income countries (LMICs).

**What did the researchers do and find?:**

- Used a microsimulation model combining vaccine impact projections with data on wealth, healthcare use, and medical/nonmedical costs across 52 Gavi-eligible countries (2000–2030).
- Estimated nearly 200 million cases of catastrophic health expenditure (CHE) averted, with nearly half benefiting the poorest quintiles.
- Identified vaccines that yield the greatest returns in deaths and CHE averted per dollar spent, providing guidance for resource allocation.

**What do these findings mean?:**

- Vaccines play a dual role: preventing illness and alleviating poverty, particularly among disadvantaged groups in LMICs.
- Sustained vaccine investments are essential to achieve health equity and poverty reduction targets, including the Sustainable Development Goals.
- A main limitation is the assumption of uniform disease risks in the absence of vaccination across quintiles, which may underestimate benefits for poorer groups.

## Abstract 3

In this modelling study, Boshen Jiao and colleagues investigate the financial risk protection vaccines can provide by preventing illnesses and associated healthcare costs in 52 low- and middle-income countries.

## Introduction

Eradicating extreme poverty by 2030 is a paramount global development objective, underscored by Sustainable Development Goal (SDG) 1 [1–3]. While macroeconomic development is crucial, large out-of-pocket (OOP) health expenditures have long been identified as a major cause of household impoverishment [4–6]. The World Health Organization (WHO) reported that governments provided on average 51% of total health spending in 2019, while more than 35% of total health spending was borne by individuals via OOP spending [6]. This situation pushes millions of individuals and families into poverty every year [7]. Financial risk protection (FRP) is central to universal health coverage (UHC); thus, the reduction in catastrophic health expenditures (CHE) has been included as a SDG target (i.e., SDG 3.8.2.) [8]. CHE are incurred when a household’s OOP health expenditures exceed a certain portion of its total income or consumption.

Beyond health and medical expenses, nonmedical costs can be significant contributors to the occurrence of CHE. For example, transportation costs incurred while seeking care and the wage losses that are associated with illness can compound the burden of OOP health expenditures and therefore increase the risk of poverty [9–11]. When combined with OOP medical costs, such nonmedical costs can impose significant financial strains on households, which highlights the multifaceted nature of the poverty-health nexus. The distributional impact of diseases is also of utmost importance, as they often disproportionately affect individuals of lower socioeconomic status, reinforcing the linkages between poor health and poverty [12–15].

To reduce poverty, OOP health expenditures and associated illness-related nonmedical costs must be mitigated by pre-payment and FRP mechanisms [16]. Importantly, on the one hand, health insurance programs can play a major role in FRP provision, notably via the public financing of expensive health interventions and procedures [17–20]. On the other hand, primary care and disease prevention programs can also improve FRP by preventing diseases and complications, and therefore the downstream financial consequences associated with the diseases they prevent. Such preventive interventions might even be more attractive in terms of efficient provision of FRP, that is, they may yield large FRP gains per given budget expenditure.

Vaccines have played a pivotal role in improving population health over the last decades [21]. A recent modeling study quantified the impact of vaccination programs, estimating the number of deaths and disability-adjusted life years (DALYs) averted through immunizations against 10 pathogens in 112 low- and middle-income countries (LMICs) from 2000 to 2030 [21,22]. The study estimated that vaccination would avert around 100 million deaths in these countries. Moreover, vaccines have the potential to provide FRP by preventing infectious diseases upstream and so the large costs associated with their treatment downstream, thus averting OOP health expenditures and potential related occurrences of CHE.

Following the COVID pandemic, the number of people living in poverty has increased [23], and vaccination rates have declined globally, exacerbating already existing public health challenges in many countries [24]. Notably, substantial human and financial resources initially dedicated to immunization programs were redirected toward responding to the COVID crisis [25]. This difficult situation poses immediate threats to public health today and emphasizes the urgency in addressing equity in vaccine distribution. It is then essential to demonstrate the potential of vaccines via the FRP benefits angle. Furthermore, the economic repercussions of COVID on LMICs have heightened poverty and social vulnerabilities [26], amplifying even more the demand for effective and efficient delivery of FRP.

In this paper, we build on a previous study exploring the equity benefits of vaccines across LMICs [27], and we estimate the distribution across socioeconomic groups of the FRP benefits provided by a range of immunizations in Gavi-eligible countries, retrospectively for the vaccination period spanning 2000–2021 and projections for vaccinations between 2022 and 2030.

## Methods

### Study design and population

We estimated the number of CHE cases averted by eight vaccines in Gavi-eligible countries, stratified by household wealth quintile. Vaccine selection was guided by two main criteria: (1) disease burden—targeting pathogens associated with high morbidity and mortality in LMICs; and (2) data coverage—focusing on vaccines for which harmonized country-specific inputs (e.g., coverage, healthcare utilization, and OOP costs) were available across at least 50 Gavi-eligible countries after merging across multiple data sources. This threshold was chosen to ensure comparability and robustness across countries. One exception was the birth dose of hepatitis B vaccine (HepB BD), which was included alongside routine HepB vaccine to provide a complete assessment of hepatitis B prevention. Eight types of vaccine doses protecting against five pathogens were considered: (1) three infant doses of hepatitis B vaccine (HepB); (2) HepB BD; (3) three infant doses of *Haemophilus influenzae* type B vaccine (Hib3); (4) three doses of *Streptococcus pneumoniae* vaccine (PCV3); (5) two or three infant doses of rotavirus vaccine (Rota); (6) first dose of measles vaccine (MCV1) delivered routinely; (7) second dose of measles vaccine (MCV2) delivered routinely; and (8) measles vaccine delivered through a campaign (SIA). Given that HepB and Hib3 can be administered jointly through pentavalent vaccine, in an additional analysis, we examined pentavalent vaccine for hepatitis B and *H. influenzae* type B prevention by aggregating the estimates of HepB and Hib3, for the countries and years, where pentavalent vaccine was administered.

We limited our analysis to countries with available estimates of disease cases prevented, costs, and healthcare utilization (S1 Table). Our analysis covered 52 countries that administered the following vaccines: HepB, HepB BD, Hib3, Rota, PCV3, MCV1, MCV2, and SIA. The target population for the analysis was the individuals vaccinated between 2000 and 2030, with historical coverage estimates from 2000 to 2021 based on WHO/UNICEF estimates of national immunization coverage (WUENIC) and other WHO/UNICEF datasets, and for future coverage projections from the Gavi operational forecast version 20 for 2022–2030 [28]. A CHE case was counted when the sum of disease-related OOP costs, transportation costs, and income losses during healthcare-seeking would exceed a certain threshold of consumption. We examined thresholds of 10, 25, and 40%, as referenced in SDG target 3.8.2. [8]. As a supplementary analysis, we also assessed impoverishing health expenditures (IHE), defined as disease-related costs that push a household below the international poverty line of $2.15 per person per day, as set by the World Bank based on 2017 purchasing power parity (PPP) [29]. IHE complements CHE by capturing the risk of absolute poverty induced by healthcare costs, rather than financial strain relative to household resources.

Additionally, we assessed the “efficiency” in delivering health benefits and FRP of several routine vaccines using two metrics: the number of deaths averted per $1 million spent; and the number of CHE cases averted per $1 million spent. For this analysis, we focused on the HepB and Hib3 component of Pentavalent, PCV3, Rota, MCV1, and MCV2, within each country. We restricted our analysis to these routine vaccines as they are administered in the majority of countries included in this study. This approach ensures a more consistent and comparable dataset for this analysis. While the pentavalent vaccine was included, it is important to note that our assessment only accounted for impact on preventing deaths and CHE related to Hib3 and HepB. The effects on diphtheria, pertussis, and tetanus were not considered, which might result in underestimations.

We used two efficiency metrics: the number of deaths averted per $1 million spent; and the number of CHE cases averted per $1 million spent.

### Data sources

Data were obtained from various sources, summarized in S2 Table. Modeled projections from the Vaccine Impact Modeling Consortium (VIMC) [21] were used for the reduction in disease cases and deaths attributable to each vaccine in each country, broken down by year of vaccination. These outputs were comprehensive and included the size of the target population, the number of individuals vaccinated, and the cases and deaths averted through vaccination over the lifetime of vaccinated birth cohorts per given country and year.

The individual-level consumption was simulated using mean consumption estimates and Gini index for each country (S3 Table). We calculated the mean consumption estimates using data from WHO’s Global Health Expenditure Database [30] and the World Bank’s World Development Indicators (WDI) for the years 2000–2021 [31]. Additionally, we used Gini index from the World Bank spanning 2000–2021 [32]. Due to gaps in consumption and Gini index data for some years, we relied on average estimates across these missing years and applied these averages for projections from 2022 to 2030.

The Decade of Vaccine Economics (DOVE) database provided cost estimates, including disease-specific per-patient healthcare utilization (inpatient and outpatient care) and the corresponding unit costs for each country based on WHO-CHOICE framework [33]. For measles, *S. pneumoniae*, *H. influenzae* type B, and rotavirus, we calculated total healthcare costs by multiplying the unit costs by the corresponding number of bed days and outpatient visits. For hepatitis B, due to the significant increase in late-stage costs in recent years, we used the average estimate from the VIMC Hepatitis B modelers and then adjusted it to reflect country-specific costs using country-specific inpatient cost data. The details can be found in S4 Table. Additionally, we calculated transportation costs and caretakers’ wage losses during healthcare seeking using data from the DOVE database. We derived proportions of OOP expenditures relative to total health expenditures based on a previous study that assessed disease-specific OOP cost shares using national health account reports [30] (S5 Table). Detailed disease-specific OOP costs for each country are available in S6 Table.

OOP costs were incurred when individuals utilized health services. Our model accounted for healthcare utilization rates, specific to each country and disease (S7 Table). However, due to the unavailability of direct data on these rates, we relied on proxy indicators derived from a comprehensive study (assuming temporal consistency) [34]. This study incorporated various sources including the Demographic and Health Surveys (DHS) [35], WHO STEPwise approach to Surveillance (STEPS) reports [36], the World Bank’s WDI [31], and Health Equity and Financial Protection Indicators (HEFPI) databases [37], as well as relevant published literature. The proxy indicators of health services utilization were disaggregated by wealth quintile. Table 1 displays summary statistics from the model inputs across countries. In summary, our model integrated consumption and health services utilization rates specific to each quintile across countries. But, due to a lack of data, the model did not differentiate disease incidence or OOP costs by quintile.

**Table 1:** Model inputs for 2000–2030 vaccinee cohorts including country-level means, medians, and interquartile ranges (IQR) across countries.

		HepB	HepB BD	Hib3	PCV3	Rota	MCV1	MCV2	SIA
Disease out-of-pocket costs (US$)^*^	Mean	251	389	69	75	44	15	15	15
	Median	217	320	60	63	36	11	11	11
	IQR	(107–359)	(251–521)	(39–97)	(42–106)	(23–64)	(8–20)	(8–19)	(8–20)
Healthcare utilization (%)	Mean	60	58	60	40	55	48	48	48
	Median	63	63	63	38	59	48	48	48
	IQR	(50–64)	(50–63)	(50–64)	(31–51)	(43–63)	(34–58)	(34–58)	(34–58)
Annual number of individuals vaccinated per country	Mean	842,000	795,000	752,000	718,000	767,000	865,000	805,000	3,688,000
	Median	276,000	132,000	263,000	265,000	277,000	263,000	239,000	1,584,000
	IQR	(97,000–670,000)	(18,600–505,000)	(97,000–673,000)	(90,000–684,000)	(103,000–630,000)	(95,000–771,000)	(87,000–599,000)	(454,000–4,552,000)
Number of disease cases averted per annual cohort per country	Mean	74,000	8,000	19,000	97,000	157,000	754,000	152,000	332,000
	Median	17,000	1,000	5,000	34,000	64,000	244,000	39,000	146,000
	IQR	(3,000–63,000)	(160–3,000)	(1,000–14,000)	(12,000–84,000)	(21,000–136,000)	(82,000–597,000)	(11,000–133,000)	(14,000–426,000)

Abbreviations: HepB: routine three infant doses of hepatitis B vaccine; HepB BD: birth dose of hepatitis B vaccine given alone; Hib3: routine three infant doses of *Haemophilus influenzae* type B vaccine; PCV3: routine three doses of *Streptococcus pneumoniae* vaccine; Rota: routine two infant doses of rotavirus vaccine; MCV1: routine first dose of measles vaccine; MCV2: routine second dose of measles vaccine; SIA: campaign measles vaccine.

*Disease out-of-pocket costs include treatment costs, multiplied by a percentage of out-of-pocket expenses, transportation costs, and wage losses during outpatient visits and hospitalizations. The country-level means for HepB and HepB BD differ due to variations in the countries where each vaccine is administered (see S1 Table).

When examining vaccination costs, we incorporated various cost factors, including the cost per vaccine dose, the cost per dose for supplies and freight, and the delivery cost per vaccine dose, all of which were sourced from the DOVE database [33]. To ensure these costs reflected the economic context of each country, they were adjusted using 2021 gross domestic product (GDP) per capita and PPP, enabling a standardized evaluation of affordability and economic burden across different settings [38].

### Modeling approach

**Fig 1:**
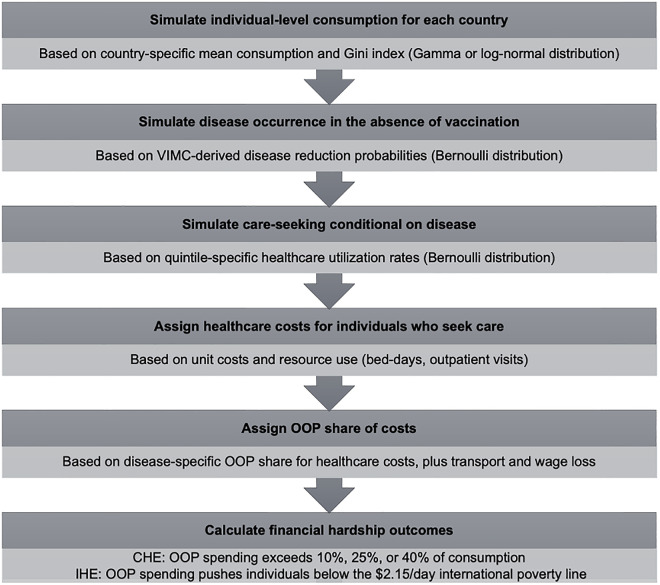
Overview of the microsimulation model for estimating financial hardship averted by vaccination. Abbreviations: CHE, catastrophic health expenditure; IHE, impoverishing health expenditure; OOP, out-of-pocket; VIMC, Vaccine Impact Modelling Consortium.

We developed a microsimulation model to estimate the number of CHE cases averted. An overview of the modeling steps is provided in Fig 1. To simulate individual-level consumption, we employed a gamma distribution based on per-capita consumption and Gini index for each country, using a sample size of *n* = 5,000. We selected the gamma distribution because it is widely used in health economics and income distribution modeling, as it flexibly represents right-skewed consumption patterns with relatively few parameters [39]. For each individual, we simulated a binary variable to indicate whether the disease, prevented by vaccine, would have occurred in the absence of vaccination: we used a Bernoulli distribution whose probability was the number of cases prevented divided by the number of vaccinated individuals, assuming constant probability across quintiles. For individuals who would have contracted the disease, we simulated a subsequent binary variable to indicate whether they would have sought medical care (also with a Bernoulli distribution). Then, we assigned costs to those individuals receiving treatment, and determined the number of CHE cases averted by immunizations based on costs, consumption, and threshold. We then calculated the average number of CHE cases averted per 10,000 vaccinated individuals from 2000 to 2030, by quintile for each vaccine. To ascertain the total number of CHE cases prevented, we multiplied this average by the overall number of individuals vaccinated in each quintile. Additionally, we conducted a region-specific analysis to explore potential differences in vaccine impact across World Bank geographic regions (e.g., Central Asia, South Asia, sub-Saharan Africa), for which the majority of countries in our study were located.

In our base-case analysis, we assumed a uniform probability of disease occurrence in the absence of vaccine across all quintiles. We conducted two sensitivity analyses to account for possible higher probabilities of diseases among lower socioeconomic groups. In the first sensitivity analysis, we adjusted the probability of disease occurrence by increasing it by 10% for the poorest and poorer quintiles, while decreasing it by 10% for the richer and richest quintiles, and maintaining the base-case probability for the middle quintile. In the second sensitivity analysis, we made a similar adjustment, increasing the probability by 20% for the poorest and poorer quintiles and decreasing it by 20% for the richer and richest quintiles. Additionally, while our base-case model simulated household consumption using a gamma distribution, we conducted a sensitivity analysis using a log-normal distribution to assess the robustness of our FRP estimates to alternative consumption specifications. Finally, we performed one-way sensitivity analyses on all parameters (±20%) to assess how variation in individual inputs affected the total number of CHE cases averted, thereby identifying the parameters most influential on the results.

In the efficiency analysis, we divided the total number of deaths averted (from VIMC model projections) and CHE cases averted by the total vaccination costs among the vaccinated individuals in each country (from DOVE). This calculation provided us with the basis for our numbers of deaths and CHE cases averted per $1 million spent. We then applied these efficiency metrics to rank routine immunizations (PCV3, Rota, MCV1, and MCV2) within each country. The pentavalent vaccine was also included, however, with only accounting for impact on preventing Hib3 and HepB. Furthermore, we derived an aggregated ranking by calculating the geometric mean of the individual rankings according to these two efficiency metrics, using a range of weights to represent a decision-maker’s preferences toward either mortality reduction or FRP.

All computations were conducted using Rstudio/2023.06.01 (www.r-project.org). This study is reported as per the CHEERS guidelines (S1 Checklist).

## Results

**Fig 2:**
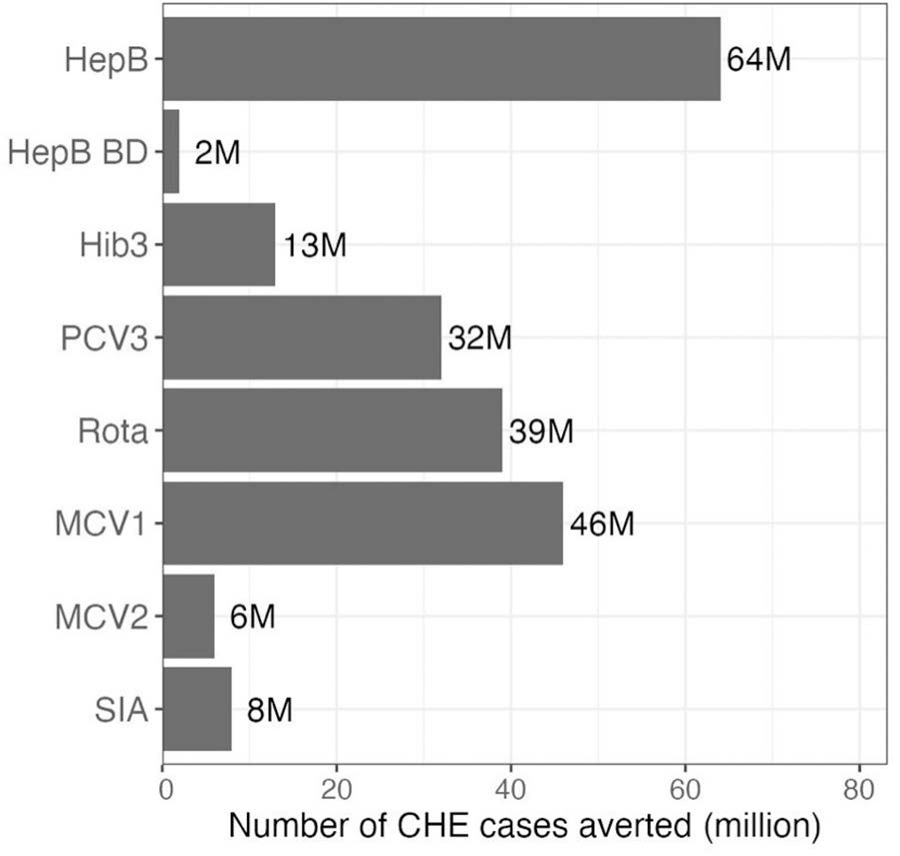
Vaccine impact on total cases of catastrophic health expenditures (CHE) averted, from 2000 to 2030 vaccinee cohorts, at a 10% CHE threshold of consumption. Abbreviations: HepB: routine three infant doses of hepatitis B vaccine; HepB BD: birth dose of hepatitis B vaccine given alone; Hib3: routine three infant doses of *Haemophilus influenzae* type B vaccine; PCV3: routine three doses of *Streptococcus pneumoniae* vaccine; Rota: routine two infant doses of rotavirus vaccine; MCV1: routine first dose of measles vaccine; MCV2: routine second dose of measles vaccine; SIA: campaign measles vaccine.

**Fig 3:**
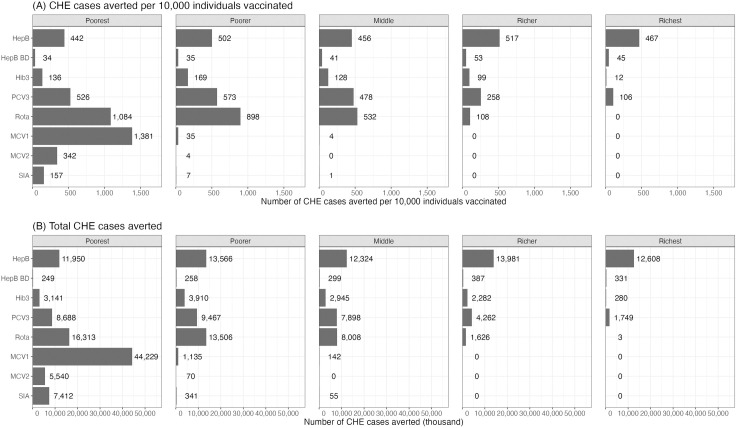
Vaccine impact on cases of catastrophic health expenditures (CHE) averted per 10,000 (A) and total cases of CHE (B), from 2000 to 2030 vaccinee cohorts, at a 10% CHE threshold of consumption. **Abbreviations:** HepB: routine three infant doses of hepatitis B vaccine; HepB BD: birth dose of hepatitis B vaccine given alone; Hib3: routine three infant doses of *Haemophilus influenzae* type B vaccine; PCV3: routine three doses of *Streptococcus pneumoniae* vaccine; Rota: routine two infant doses of rotavirus vaccine; MCV1: routine first dose of measles vaccine; MCV2: routine second dose of measles vaccine; SIA: campaign measles vaccine.

We first present the FRP benefits for all studied immunizations (Fig 2 and S8 Table), showing a substantial collective impact. Across the 2000–2030 vaccinee cohorts, ~200 million CHE cases would be averted at the 10% threshold. By vaccine, the HepB vaccine prevents 64 million cases of CHE, followed by MCV1 with 46 million cases, Rota with 39 million cases, and PCV3 with 32 million cases averted. Additionally, we display the FRP benefits by wealth quintile (Fig 3), highlighting a greater impact among lower wealth groups. MCV1 demonstrated the highest number of CHE cases averted per 10,000 vaccinated individuals in the poorest quintile, followed by Rota, PCV3, HepB, MCV2, SIA, Hib3, and HepB BD. In terms of total CHE cases averted, MCV1 and Rota remained the highest-ranking vaccines for the poorest quintile, followed by HepB, PCV3, SIA, MCV2, Hib3, and HepB BD. Conversely, among the richest quintile, HepB contributed to the greatest number of CHE cases averted, followed by PCV3, HepB BD, and Hib3, with minimal to no measurable impact observed for other vaccines. Notably, at the 10% threshold, the impact of HepB and HepB BD remained consistent across quintiles, as the high disease cost can be a financial burden for both the poorest and richest groups. Finally, when considering the combined impact of HepB and Hib3 as a pentavalent vaccine, it ranked third among the poorest quintile, following MCV1 and Rota, but ranked first among the richest quintile (S1 Fig).

S2 and S3 Figs depict the number of CHE cases averted from 2000 to 2021 and projections for 2022–2030, respectively. The number of CHE cases averted per 10,000 would align closely with the estimates for the 2000–2030 period mentioned earlier. However, when examining total CHE cases averted, vaccines display varied trends between these two time periods. For instance, recently introduced vaccines (e.g., Rota, PCV3) are projected to have a sharp increase in CHE cases averted from 2022 to 2030 (compared to 2000–2021), due to their later introduction. In contrast, the impact of MCV1 is anticipated to diminish in the 2022–2030 period. In addition, S4 and S5 Figs present the number of CHE cases averted with alternative thresholds of 25% and 40%. Expectedly, at these higher thresholds, the CHE cases averted would be considerably lower than at the 10% threshold. For IHE, vaccines such as MCV1, MCV2, and SIA avert substantially more cases than under the CHE metric, particularly among the poorest and poorer quintiles (S6 Fig). Furthermore, S7 Fig shows results for selected geographic regions. When examining the number of CHE cases averted per 10,000 vaccinated, for HepB, HepB BD, MCV1, and SIA, the largest impact was observed in sub-Saharan Africa; while Hib3, PCV3 Rota, and MCV2 demonstrated their greatest impact in South Asia.

In the sensitivity analyses where we varied the distributional probability of disease occurrence across wealth quintiles (S8 and S9 Figs), disparities in FRP outcomes became more pronounced. The poorest and poorer quintiles experienced even greater gains in FRP, reflecting the higher adjusted probabilities of disease in these groups. When using a log-normal distribution for consumption, similar patterns emerged, although CHE cases averted slightly increased among upper quintiles and decreased among lower quintiles compared to the gamma distribution (S10 Fig). In the one-way sensitivity analyses of all parameters, the Gini index emerged as the most influential factor, with higher inequality leading to more CHE cases averted—indicating that greater income disparity amplifies the FRP benefits of vaccination (S11 Fig).

Table 2 shows the distribution of vaccines (across countries), categorized by their ranking based on the number of deaths and CHE cases (10% threshold) averted per $1 million spent. In the majority of countries, MCV1 was ranked first with respect to deaths averted per $1 million spent, while Rota was typically ranked fifth. In terms of CHE cases averted per $1 million spent, MCV1 would occupy the top ranking in most countries, whereas MCV2 and PCV3 would often be ranked fifth.

**Table 2:** Percentage of countries with specific vaccines in each ranking position, according to either catastrophic health expenditures averted (CHE, at a threshold of 10% of consumption) (left panel) or deaths averted (right panel), per $1 million spent.

CHE Averted per $1M			Deaths Averted per $1M		
Rank	Vaccine	% of countries with vaccine at this rank	Rank	Vaccine	% of countries with vaccine at this rank
1	MCV1	56%	1	MCV1	96%
	Penta	29%		Penta	4%
	Rota	13%	2	MCV2	58%
	PCV3	2%		Penta	38%
2	Penta	38%		MCV1	4%
	Rota	35%	3	Penta	56%
	MCV1	25%		MCV2	38%
	PCV3	2%		PCV3	6%
3	Rota	37%	4	PCV3	90%
	Penta	23%		Rota	8%
	MCV2	19%		Penta	2%
	MCV1	15%	5	Rota	96%
	PCV3	6%		PCV3	4%
4	PCV3	52%			
	MCV2	29%			
	Rota	10%			
	Penta	6%			
	MCV1	4%			
5	MCV2	50%			
	PCV3	40%			
	Rota	6%			
	Penta	4%			

Abbreviations: PCV3: routine three doses of *Streptococcus pneumoniae* vaccine; Rota: routine two infant doses of rotavirus vaccine; MCV1: routine first dose of measles vaccine; MCV2: routine second dose of measles vaccine; SIA: campaign measles vaccine; Penta: pentavalent vaccine for prevention of hepatitis B and *Haemophilus influenzae* type.

S12 Fig displays the percentage of countries with specific vaccines in each aggregated ranking, across preference weights ranging from 0 to 1 for CHE averted, where 0 indicates no preference towards CHE averted and 1 indicates no preference towards deaths averted. MCV1 and pentavalent vaccines would consistently occupy top ranks over the entire range of preferences. Rota would become the third optimal choice when prioritizing averting CHE over deaths.

## Discussion

We conducted an analysis of the FRP provided by vaccines targeting hepatitis B, *H. influenzae* type B, rotavirus, measles, and *S. pneumoniae*. Our findings suggest that these immunization efforts could avert ~200 million cases of CHE across 52 Gavi-eligible countries from 2000 to 2030, using a 10% consumption threshold. Notably, nearly half of these averted cases would benefit individuals in the poorest quintile.

Our analysis utilized projections of the number of disease cases averted by various disease models for 52 LMICs. We incorporated various secondary data sources, including on health services utilization following disease onset, direct costs associated with treatment of vaccine-preventable diseases, and the transportation costs and wage losses when seeking healthcare for these diseases. We evaluated the FRP outcomes across wealth quintiles in these countries and analyzed the potential efficiency in terms of both FRP and averting deaths per budget expenditure.

Our findings reveal significant variations in the magnitude of FRP provided by the different vaccines. For instance, MCV1 could avert as many as 44 million CHE cases among the poorest, compared with 7 million cases for campaign measles vaccines. Although the lower treatment costs for measles might result in a smaller CHE impact, MCV1 would still prevent a large number of CHE cases by averting a substantial number of measles cases. Conversely, campaign measles vaccines avert fewer disease cases compared to MCV1 (due to sporadic implementations), leading to a lesser number of CHE cases prevented. It is important to recognize that the CHE and disease cases averted by subsequent vaccinations are contingent on the coverage achieved by preceding ones, as well as the specific epidemiological context of each country. For example, in countries with scant MCV1 coverage, SIA for measles is likely to see a greater impact, averting more cases and associated CHE. In Gavi-supported countries that have introduced the HepB BD, coverage remains minimal, with India representing a significant share of immunized children. Given India’s lower to intermediate prevalence and reduced perinatal transmission rates, the HepB BD prevents fewer cases than it would in African nations.

Furthermore, the CHE cases averted would vary across different wealth quintiles. In general, the number of CHE cases averted decreased with wealth: the same amount of OOP spending represents a higher proportion of consumption among poorer households than wealthier ones. While this could be mitigated by lower health services utilization among poorer individuals, our model accounts for this and suggests that it does not for most vaccines. Notably, HepB and HepB BD demonstrated similar impacts across wealth quintiles at the 10% threshold, as the high disease costs represent a substantial burden for both poorer and wealthier households. However, at higher thresholds, such as 25% or 40%, these costs would be catastrophic for most poorer households, while the relative burden would be less pronounced for wealthier groups. Similarly, the regional distribution of CHE cases averted shows variations. For instance, MCV1 would demonstrate the highest impact in sub-Saharan Africa, whereas Rota would be most effective in South Asia. These differences result from the differential expected health impact of vaccines across regions and are also influenced by heterogeneities in health services utilization, costs, and consumption levels.

In addition to CHE, we examined IHE as a complementary indicator of FRP. While pro-poor patterns were observed across vaccines, MCV1, MCV2, and SIA demonstrated substantially greater impact under the IHE metric, particularly among the lower quintiles. This reflects the nature of measles-related treatment costs, which, although often too low to exceed the CHE threshold, may still be sufficient to push low-income households below the international poverty line. These findings underscore the importance of incorporating multiple FRP metrics—such as IHE—into future evaluations, as they can capture additional dimensions of vaccine impact that are especially relevant to equity-focused policy efforts.

Efforts to reduce extreme poverty by 2030 are underway. However, according to World Bank 2019 data, there were ~1 billion CHE cases worldwide [40]. Our study thus highlights the potential of expanding vaccination programs to make a substantial contribution towards reducing poverty, particularly among the most impoverished in LMICs.

It is worth noting the growing emphasis on equity in economic evaluation, particularly through emerging frameworks such as distributional cost-effectiveness analysis (DCEA) and extended cost-effectiveness analysis (ECEA) [41–43]. Our work is complementary to recent advances in DCEA [41,43], which incorporate inequality aversion directly into the valuation of health gains. In contrast, our analysis emphasizes economic vulnerability by focusing on financial hardship rather than disparities in health outcomes. At the same time, our analysis aligns closely with the principles of ECEA [42], which explicitly incorporate both FRP and equity considerations into cost-effectiveness frameworks. Together, these approaches reflect a broader shift toward equity-informed priority-setting in global health.

We also found that MCV1 and the pentavalent vaccine would likely generate more returns in terms of deaths and CHE cases averted per budget expenditure. These vaccines would remain most favorable across a range of decision-making preferences across the mortality reduction and FRP dimensions. This is not solely attributable to their robust ability to save lives and avert CHE cases, but also to their relatively lower procurement costs. Conversely, Rota and PCV3 would show lower returns per budget expenditure. This is primarily due to the higher costs associated with these immunizations. While our efficiency analysis was not stratified by wealth quintile due to data limitations on mortality by socioeconomic group, future work exploring equity-efficiency tradeoffs could inform more targeted prioritization strategies.

Importantly, it is crucial to note that financing for immunization programs must include additional investments in health system strengthening to achieve the desired health outcomes. Taking the example of Zambia, despite high MCV1 coverage, repeated measles outbreaks can occur due to clusters of unvaccinated individuals [44]. These groups might either lack connection to the health system or harbor distrust toward it. Therefore, vaccine financing should be paired with initiatives to bolster health system strengthening, ensuring vaccine accessibility for the vulnerable and enhancing trust in the health system.

This study offers several key strengths, including broad geographic coverage, methodological rigor, and an explicit focus on equity. By incorporating standardized inputs across 52 Gavi-eligible countries, it provides one of the most extensive cross-country assessments of the FRP benefits of vaccination in low- and middle-income settings. The microsimulation approach allows for heterogeneity in consumption levels and care-seeking behavior by wealth quintile, enabling detailed analysis of the distributional effects of vaccines. Additionally, the inclusion of CHE and IHE metrics offers a comprehensive understanding of how vaccines protect households from financial hardship. Finally, the integration of FRP metrics with efficiency analysis supports policy-relevant insights for priority setting in resource-constrained environments.

Nevertheless, this study presents several limitations, with important data constraints. First, while we have incorporated parameters varying by quintile, such as consumption and health services utilization, we lacked quintile-specific information on disease risk in the absence of vaccination, as well as detailed OOP health and nonhealth costs differentiated by quintile. As a result, we made simplifying assumptions, such as crudely assuming constant disease risks and OOP costs across quintiles. It should be recognized though that individuals within lower wealth quintiles often exhibit higher risks for those infectious diseases; thus, in scenarios where vaccines or treatments are unavailable, there could be greater disease occurrence within these segments of the population [13–15]. This limitation may thus have led to an underestimation of the FRP benefits for the lower quintiles, as demonstrated in sensitivity analyses (S7 and S8 Figs). We also used proxy indicators for health services utilization, which might not reflect actual care-seeking behaviors and utilization patterns across different socioeconomic groups. Moreover, our disease cost estimates were derived from DOVE databases, relying on average figures rather than detailed cost inputs that account for the diversity in disease presentations and include recent advanced treatments. For example, while our estimates for hepatitis B encompass healthcare and nonhealth expenses for both chronic and acute symptomatic cases, they may not fully capture the costs of advanced long-term treatments necessary for managing chronic conditions and their complications. Consequently, our cost estimates tend to be conservative. In addition, for countries with missing consumption and Gini index data, we imputed average values across available years. This likely understates temporal variation in inequality—especially in countries experiencing economic shocks or rising inequality—thereby flattening the equity gradient and underestimating the relative vulnerability of poorer households. Finally, our analysis incorporated parameter inputs from multiple external sources (e.g., VIMC, DOVE), which, although representing the best available inputs, may introduce additional uncertainty into our estimates.

Second, our analysis relied on distinct disease models for each vaccine impact; thus, we were not able to account for competing risks among the same individuals [21,22]. However, the influence of this factor on our findings might be small as previously pointed by Li and colleagues [22]. Third, our modeling approach was unable to account for cases where individuals would experience successive and repeated episodes of diseases over time, along with OOP expenses occurring within the same household. Similarly, while we assumed that individuals not seeking care upon infection incurred no immediate OOP costs, we overlooked the potential for these untreated cases to lead to complications or sequelae resulting in OOP costs in the future. Including these potential costs would likely increase our estimate of the FRP impact of vaccines. Fourth, there are inherent limitations when using CHE indicators. Such metrics are susceptible to variations based on the selected thresholds that influence the results. Additionally, while FRP metrics provide valuable insights into the financial aspects of healthcare, they fail to capture other essential considerations for priority setting, including access improvement and health system strengthening. Fifth, our study included vaccines against five pathogens based on both disease burden and data availability. Expanding the analysis to include additional vaccine-preventable diseases could increase the overall estimated FRP benefits, particularly for older age groups and adolescent populations. However, due to gaps in disease-specific data across all countries for these additional vaccines, we were unable to include them here. As a result, our findings should be interpreted as estimates of the FRP benefits of key childhood vaccines, rather than of immunization programs as a whole. Sixth, the estimated averted CHE cases reported in our study represent future benefits over the lifetime of cohorts vaccinated between 2000 and 2030. We did not discount these future benefits or incorporate changes in simulated household consumptions, OOP costs, or transport costs, which might be important for the long-term impact of immunizations like Hepatitis B.

Vaccinations are known to have significant mortality and morbidity reduction benefits and are efficient interventions [45]. Our study further demonstrates the strong potential of vaccines for providing FRP, particularly among disadvantaged populations and the poorest. Given the pressing priorities of poverty reduction and equity improvement globally, it is imperative to make sustained investments toward vaccines in LMICs. By doing so, we can make a significant contribution towards achieving the SDGs and UHC, ensuring a healthier and more equitable future for all.

## Supporting information

S1 Table List of countries analyzed for each vaccine in the study. (DOCX)

S2 Table Summary of model parameters and data sources. (DOCX)

S3 Table Country-specific mean consumption and Gini index are used to simulate individual-level consumption. (DOCX)

S4 Table Inputs for healthcare utilization and treatment costs are used in country-specific cost calculations. (DOCX)

S5 Table Country-specific out-of-pocket shares for vaccine-preventable diseases. (DOCX)

S6 Table Country-specific out-of-pocket costs for vaccine-preventable diseases. (DOCX)

S7 Table Country- and quintile-specific care-seeking rates conditional on disease occurrence. (DOCX)

S8 Table Vaccine impact on cases of catastrophic health expenditures (CHE) and impoverishing health expenditure (IHE) averted per 10,000 and total cases of CHE, from 2000 to 2030 vaccinee cohorts. (DOCX)

S1 Fig Vaccine impact including pentavalent vaccine (HepB and Hib3) on cases of catastrophic health expenditures (CHE) averted per 10,000 (A) and total cases of CHE (B), from 2000 to 2030 vaccinee cohorts, at a 10% CHE threshold of consumption. (DOCX)

S2 Fig Vaccine impact on cases of catastrophic health expenditures (CHE) averted per 10,000 (A) and total cases of CHE (B), from 2000 to 2021 vaccinee cohorts, at a 10% CHE threshold of consumption. (DOCX)

S3 Fig Vaccine impact on cases of catastrophic health expenditures (CHE) averted per 10,000 (A) and total cases of CHE (B), from 2022 to 2030 vaccinee cohorts at a 10% CHE threshold of consumption. (DOCX)

S4 Fig Vaccine impact on cases of catastrophic health expenditure (CHE) averted from 2000 to 2030 vaccinee cohorts, at a 25% CHE threshold of consumption. (DOCX)

S5 Fig Vaccine impact on cases of catastrophic health expenditure (CHE) averted from 2000 to 2030 vaccinee cohorts, at a 40% CHE threshold of consumption. (DOCX)

S6 Fig Vaccine impact on cases of impoverishing health expenditures (IHE) averted among 2000–2030 vaccinee cohorts, using a $2.15-per-day poverty threshold. (DOCX)

S7 Fig Vaccine impact of immunizations on cases of catastrophic health expenditure (CHE) averted from 2000 to 2030 vaccinee cohorts by geographic region, at a 10% CHE threshold of consumption. (DOCX)

S8 Fig Sensitivity analysis results: adjusted disease probability without immunization by wealth quintile—a 10% increase for the poorest and poorer, and a 10% decrease for the richest and richer, at a 10% catastrophic health expenditures (CHE) threshold of consumption. (DOCX)

S9 Fig Sensitivity analysis results: adjusted disease probability without immunization by wealth quintile—a 20% increase for the poorest and poorer, and a 20% decrease for the richest and richer, at a 10% catastrophic health expenditures (CHE) threshold of consumption. (DOCX)

S10 Fig Sensitivity analysis: using a log-normal distribution to simulate individual-level consumption. (DOCX)

S11 Fig One-way sensitivity analysis of key parameters on total catastrophic health expenditures (CHE) averted. (DOCX)

S12 Fig Percentage of countries with specific vaccines in each aggregated ranking over a range of preference weights (from 0 to 1) for catastrophic health expenditures (CHE). (DOCX)

S1 Checklist Consolidated Health Economic Evaluation Reporting Standards 2022 (CHEERS 2022). (DOCX)

## Abbreviations

CHE: catastrophic health expenditure
DALYs: disability-adjusted life years
DCEA: distributional cost-effectiveness analysis
DHS: Demographic and Health Surveys
DOVE: Decade of Vaccine Economics
ECEA: extended cost-effectiveness analysis
FRP: financial risk protection
GDP: gross domestic product
HEFPI: Health Equity and Financial Protection Indicators
IHE: impoverishing health expenditures
IQR: interquartile range
LMICs: low- and middle-income countries
OOP: out-of-pocket
PPP: purchasing power parity
SDG: Sustainable Development Goal
STEPS: STEPwise approach to Surveillance
UHC: universal health coverage
VIMC: Vaccine Impact Modeling Consortium
WDI: World Development Indicators
WHO: World Health Organization
WUENIC: WHO/UNICEF estimates of national immunization coverage
