# Supplementary material for: Financial risk protection from vaccines in 52 Gavi-eligible low- and middle-income countries: A modeling study
Source: PLoS Med. 2025 Nov 4;22(11):e1004764. doi: 10.1371/journal.pmed.1004764 (PMC12585062; doi:10.1371/journal.pmed.1004764)
Supplement: S1 Table — (DOCX) [file pmed.1004764.s001.docx]

**S1 Table. List of countries analyzed for each vaccine in the study.**

| **Country** | **HepB** | **HepB BD** | **Hib3** | **PCV3** | **Rota** | **MCV1** | **MCV2** | **SIA** |
| --- | --- | --- | --- | --- | --- | --- | --- | --- |
| Bangladesh | X | X | X | X | X | X | X | X |
| Benin | X | X | X | X | X | X | X | X |
| Burkina Faso | X | X | X | X | X | X | X | X |
| Burundi | X | X | X | X | X | X | X | X |
| Cameroon | X | X | X | X | X | X | X | X |
| Central African Republic | X | X | X | X | X | X | X | X |
| Chad | X | X | X | X | X | X | X | X |
| Congo | X | X | X | X | X | X | X | X |
| Congo, the Democratic Republic of the | X | X | X | X | X | X | X | X |
| Cote d'Ivoire | X | X | X | X | X | X | X | X |
| Ethiopia | X | X | X | X | X | X | X | X |
| Ghana | X | X | X | X | X | X | X | X |
| Guinea | X | X | X | X | X | X | X | X |
| Guinea-Bissau | X | X | X | X | X | X | X | X |
| Haiti | X | X | X | X | X | X | X | X |
| India | X |  | X | X | X | X | X | X |
| Kenya | X |  | X | X | X | X | X | X |
| Liberia | X |  | X | X | X | X | X | X |
| Malawi | X |  | X | X | X | X | X | X |
| Mali | X |  | X | X | X | X | X | X |
| Mauritania | X |  | X | X | X | X | X | X |
| Mozambique | X |  | X | X | X | X | X | X |
| Nepal | X |  | X | X | X | X | X | X |
| Niger | X |  | X | X | X | X | X | X |
| Nigeria | X |  | X | X | X | X | X | X |
| Pakistan | X |  | X | X | X | X | X | X |
| Sierra Leone | X |  | X | X | X | X | X | X |
| Somalia | X |  | X | X | X | X | X | X |
| South Sudan | X |  | X | X | X | X | X | X |
| Tanzania, United Republic of | X |  | X | X | X | X | X | X |
| Togo | X |  | X | X | X | X | X | X |
| Uganda | X |  | X | X | X | X | X | X |
| Yemen | X |  | X | X | X | X | X | X |
| Zambia | X |  | X | X | X | X | X | X |
| Zimbabwe | X |  | X | X | X | X | X | X |
| Comoros | X |  | X | X | X | X | X | X |
| Djibouti | X |  | X | X | X | X | X | X |
| Gambia | X |  | X | X | X | X | X | X |
| Kyrgyzstan | X |  | X | X | X | X | X | X |
| Lao People's Democratic Republic | X |  | X | X | X | X | X | X |
| Lesotho | X |  | X | X | X | X | X | X |
| Madagascar | X |  | X | X | X | X | X | X |
| Myanmar | X |  | X | X | X | X | X | X |
| Nicaragua | X |  | X | X | X | X | X | X |
| Papua New Guinea | X |  | X | X | X | X | X | X |
| Rwanda | X |  | X | X | X | X | X | X |
| Sao Tome and Principe | X |  | X | X | X | X | X | X |
| Senegal | X |  | X | X | X | X | X | X |
| Solomon Islands | X |  | X | X | X | X | X | X |
| Sudan | X |  | X | X | X | X | X | X |
| Tajikistan | X |  | X | X | X | X |  | X |
| Uzbekistan | X |  | X | X | X | X |  | X |

HepB: routine three infant doses of hepatitis B vaccine; HepB BD: birth dose of hepatitis B vaccine given alone; Hib3: routine three infant doses of *Haemophilus influenzae* type B vaccine; PCV3: routine three doses of *Streptococcus pneumoniae* vaccine; Rota: routine two infant doses of rotavirus vaccine; MCV1: routine first dose of measles vaccine; MCV2: routine second dose of measles vaccine; SIA: campaign measles vaccine.
