# Supplementary material for: Financial risk protection from vaccines in 52 Gavi-eligible low- and middle-income countries: A modeling study
Source: PLoS Med. 2025 Nov 4;22(11):e1004764. doi: 10.1371/journal.pmed.1004764 (PMC12585062; doi:10.1371/journal.pmed.1004764)
Supplement: S2 Table — (DOCX) [file pmed.1004764.s002.docx]

**S2 Table. Summary of model parameters and data sources**

| **Model component** | **Parameter/description** | **Data source** | **Statistical assumption** |
| --- | --- | --- | --- |
| Individual consumption | Simulated from mean consumption and Gini index per country | WHO Global Health Expenditure Database, WDI, World Bank | Gamma or log-normal distribution (n=5000) |
| Disease occurrence | Probability of disease occurrence in absence of vaccine | VIMC | Bernoulli distribution |
| Care-seeking behavior | Utilization rates by wealth quintile and disease | Bolongaita et al., DHS, WHO STEPS, HEFPI, published literature | Bernoulli distribution |
| Healthcare costs | Unit cost × resource use (bed days, outpatient visits) | DOVE, VIMC, literature | Deterministic assignment based on observed averages |
| OOP share of costs | Disease-specific % of OOP | National Health Account reports | Fixed proportion per disease-country |
| Financial hardship outcomes | CHE: OOP >10%, 25%, or 40% of consumption; IHE: falls below $2.15/day poverty line | WHO SDG Indicator 3.8.2, World Bank poverty line | Threshold-based indicator |
| Deaths averted | Lifetime deaths averted by vaccine and country | VIMC | Deterministic from VIMC projections |
| Vaccination coverage | Vaccine coverage by year and country (2000–2030) | WUENIC, Gavi Operational Forecast v20 | Deterministic from administrative projections |
| Vaccination costs | Vaccine cost + supply/freight + delivery cost | DOVE | Adjusted by GDP per capita and PPP |

CHE: Catastrophic Health Expenditure; DHS: Demographic and Health Surveys; DOVE: Decade of Vaccine Economics; GDP: Gross Domestic Product; GNI: Gross National Income; HEFPI: Health Equity and Financial Protection Indicators (HEFPI); IHE: Impoverishing Health Expenditure; OOP: Out-of-pocket; PCV: Pneumococcal Conjugate Vaccine; PPP: Purchasing Power Parity; VIMC: Vaccine Impact Modeling Consortium; WHO: World Health Organization; WHO STEPS: WHO STEPwise approach to Surveillance; WUENIC: WHO/UNICEF Estimates of National Immunization Coverage.
