# Supplementary material for: Financial risk protection from vaccines in 52 Gavi-eligible low- and middle-income countries: A modeling study
Source: PLoS Med. 2025 Nov 4;22(11):e1004764. doi: 10.1371/journal.pmed.1004764 (PMC12585062; doi:10.1371/journal.pmed.1004764)
Supplement: S3 Table — (DOCX) [file pmed.1004764.s003.docx]

**S3 Table. Country-specific mean consumption and Gini index used to simulate individual-level consumption**

| **Country** | **Mean consumption** | **Gini index** |
| --- | --- | --- |
| Bangladesh | 807 | 0.328 |
| Benin | 785 | 0.419 |
| Burkina Faso | 441 | 0.414 |
| Burundi | 182 | 0.360 |
| Cameroon | 898 | 0.438 |
| Central African Republic | 344 | 0.562 |
| Chad | 540 | 0.402 |
| Comoros | 1243 | 0.506 |
| Congo | 797 | 0.481 |
| Congo, the Democratic Republic of the | 343 | 0.422 |
| Cote d'Ivoire | 1189 | 0.408 |
| Djibouti | 1234 | 0.427 |
| Ethiopia | 352 | 0.327 |
| Gambia | 566 | 0.423 |
| Ghana | 1141 | 0.429 |
| Guinea | 593 | 0.364 |
| Guinea-Bissau | 495 | 0.404 |
| Haiti | 1165 | 0.411 |
| India | 826 | 0.352 |
| Kenya | 972 | 0.437 |
| Kyrgyzstan | 841 | 0.300 |
| Lao People's Democratic Republic | 1018 | 0.357 |
| Lesotho | 830 | 0.483 |
| Liberia | 682 | 0.350 |
| Madagascar | 330 | 0.431 |
| Malawi | 285 | 0.422 |
| Mali | 510 | 0.370 |
| Mauritania | 892 | 0.369 |
| Mozambique | 350 | 0.489 |
| Myanmar | 508 | 0.344 |
| Nepal | 607 | 0.383 |
| Nicaragua | 1257 | 0.480 |
| Niger | 325 | 0.370 |
| Nigeria | 1442 | 0.365 |
| Pakistan | 857 | 0.299 |
| Papua New Guinea | 1042 | 0.419 |
| Rwanda | 472 | 0.473 |
| Sao Tome and Principe | 1079 | 0.345 |
| Senegal | 936 | 0.397 |
| Sierra Leone | 454 | 0.366 |
| Solomon Islands | 1191 | 0.416 |
| Somalia | 553 | 0.368 |
| South Sudan | 410 | 0.452 |
| Sudan | 897 | 0.348 |
| Tajikistan | 703 | 0.327 |
| Tanzania, United Republic of | 492 | 0.390 |
| Togo | 474 | 0.434 |
| Uganda | 521 | 0.431 |
| Uzbekistan | 1072 | 0.348 |
| Yemen | 914 | 0.357 |
| Zambia | 656 | 0.527 |
| Zimbabwe | 1180 | 0.459 |
