# Supplementary material for: Financial risk protection from vaccines in 52 Gavi-eligible low- and middle-income countries: A modeling study
Source: PLoS Med. 2025 Nov 4;22(11):e1004764. doi: 10.1371/journal.pmed.1004764 (PMC12585062; doi:10.1371/journal.pmed.1004764)
Supplement: S4 Table — (DOCX) [file pmed.1004764.s004.docx]

**S4 Table. Inputs for healthcare utilization and treatment costs used in country-specific cost calculations**

| **Antigen** | **Healthcare utilization or costs** |
| --- | --- |
| Measles | Average inpatient bed days: 1.33 Average outpatient visits per case: 0.5 |
| *Streptococcus pneumoniae* | Average pneumonia inpatient bed days: 6 Average meningitis Inpatient bed days: 13 Average proportion of pneumonia cases: 70% |
| *Haemophilus influenzae* type B | Average pneumonia inpatient bed days: 6 Average meningitis Inpatient bed days: 13 Average proportion of pneumonia cases: 72% |
| Rotavirus | Average inpatient bed days: 4.25 |
| Hepatitis B* | Average late-stage liver disease cost per chronic carrier per year: $909 Average cost of treatment per chronic carrier per year: $4 |

*The average treatment costs for Hepatitis B were derived from Hepatitis B modelers, incorporating late-stage costs. These costs were then adjusted to reflect country-specific expenses using inpatient cost data from the DOVE database.
