# Supplementary material for: Financial risk protection from vaccines in 52 Gavi-eligible low- and middle-income countries: A modeling study
Source: PLoS Med. 2025 Nov 4;22(11):e1004764. doi: 10.1371/journal.pmed.1004764 (PMC12585062; doi:10.1371/journal.pmed.1004764)
Supplement: S5 Table — (DOCX) [file pmed.1004764.s005.docx]

**S5 Table. Country-specific out-of-pocket shares for vaccine-preventable diseases**

| **Country** | **HepB** | **Hib** | **PCV** | **Rota** | **Measles** |
| --- | --- | --- | --- | --- | --- |
| Bangladesh | 0.63 | 0.71 | 0.71 | 0.86 | 0.45 |
| Benin | 0.64 | 0.71 | 0.71 | 0.66 | 0.42 |
| Burkina Faso | 0.64 | 0.71 | 0.71 | 0.66 | 0.42 |
| Burundi | 0.37 | 0.40 | 0.40 | 0.48 | 0.39 |
| Cameroon | 0.21 | 0.44 | 0.44 | 0.37 | 0.44 |
| Central African Republic | 0.21 | 0.44 | 0.44 | 0.37 | 0.45 |
| Chad | 0.21 | 0.44 | 0.44 | 0.37 | 0.45 |
| Comoros | 0.41 | 0.44 | 0.44 | 0.42 | 0.39 |
| Congo, the Democratic Republic of the | 0.17 | 0.58 | 0.58 | 0.74 | 0.45 |
| Congo | 0.23 | 0.50 | 0.50 | 0.28 | 0.46 |
| Cote d'Ivoire | 0.79 | 0.92 | 0.92 | 0.92 | 0.42 |
| Djibouti | 0.41 | 0.44 | 0.44 | 0.42 | 0.39 |
| Ethiopia | 0.41 | 0.44 | 0.44 | 0.42 | 0.52 |
| Gambia | 0.42 | 0.71 | 0.71 | 0.66 | 0.42 |
| Ghana | 0.68 | 0.60 | 0.60 | 0.50 | 0.42 |
| Guinea | 0.74 | 0.51 | 0.51 | 0.75 | 0.42 |
| Guinea-Bissau | 0.64 | 0.71 | 0.71 | 0.66 | 0.42 |
| Haiti | 0.59 | 0.52 | 0.52 | 0.73 | 0.45 |
| India | 0.63 | 0.71 | 0.71 | 0.86 | 0.45 |
| Kenya | 0.16 | 0.31 | 0.31 | 0.31 | 0.39 |
| Kyrgyzstan | 0.56 | 0.58 | 0.58 | 0.70 | 0.45 |
| Lao People's Democratic Republic | 0.78 | 0.41 | 0.41 | 0.59 | 0.45 |
| Lesotho | 0.09 | 0.57 | 0.57 | 0.53 | 0.42 |
| Liberia | 0.64 | 0.71 | 0.71 | 0.66 | 0.42 |
| Madagascar | 0.41 | 0.44 | 0.44 | 0.42 | 0.39 |
| Malawi | 0.15 | 0.44 | 0.44 | 0.07 | 0.39 |
| Mali | 0.72 | 0.73 | 0.73 | 0.49 | 0.42 |
| Mauritania | 0.64 | 0.71 | 0.71 | 0.66 | 0.42 |
| Mozambique | 0.72 | 0.91 | 0.91 | 0.42 | 0.39 |
| Myanmar | 0.84 | 0.88 | 0.88 | 0.85 | 0.45 |
| Nepal | 0.63 | 0.71 | 0.71 | 0.86 | 0.39 |
| Nicaragua | 0.36 | 0.41 | 0.41 | 0.73 | 0.45 |
| Niger | 0.71 | 0.66 | 0.66 | 0.76 | 0.42 |
| Nigeria | 0.78 | 0.96 | 0.96 | 0.79 | 0.42 |
| Pakistan | 0.63 | 0.71 | 0.71 | 0.86 | 0.45 |
| Papua New Guinea | 0.25 | 0.44 | 0.44 | 0.55 | 0.45 |
| Rwanda | 0.41 | 0.44 | 0.44 | 0.42 | 0.39 |
| Sao Tome and Principe | 0.24 | 0.23 | 0.23 | 0.09 | 0.45 |
| Senegal | 0.66 | 0.61 | 0.61 | 0.52 | 0.42 |
| Sierra Leone | 0.64 | 0.71 | 0.71 | 0.66 | 0.42 |
| Solomon Islands | 0.25 | 0.44 | 0.44 | 0.55 | 0.45 |
| Somalia | 0.41 | 0.44 | 0.44 | 0.42 | 0.39 |
| South Sudan | 0.41 | 0.44 | 0.44 | 0.42 | 0.39 |
| Sudan | 0.47 | 0.57 | 0.57 | 0.53 | 0.42 |
| Tajikistan | 0.56 | 0.58 | 0.58 | 0.70 | 0.45 |
| Tanzania, United Republic of | 0.41 | 0.44 | 0.44 | 0.42 | 0.27 |
| Togo | 0.64 | 0.71 | 0.71 | 0.66 | 0.42 |
| Uganda | 0.55 | 0.41 | 0.41 | 0.54 | 0.39 |
| Uzbekistan | 0.56 | 0.58 | 0.58 | 0.70 | 0.45 |
| Yemen | 0.82 | 0.24 | 0.24 | 0.46 | 0.39 |
| Zambia | 0.41 | 0.44 | 0.44 | 0.42 | 0.39 |
| Zimbabwe | 0.41 | 0.44 | 0.44 | 0.42 | 0.39 |
