# Supplementary material for: Financial risk protection from vaccines in 52 Gavi-eligible low- and middle-income countries: A modeling study
Source: PLoS Med. 2025 Nov 4;22(11):e1004764. doi: 10.1371/journal.pmed.1004764 (PMC12585062; doi:10.1371/journal.pmed.1004764)
Supplement: S6 Table — (DOCX) [file pmed.1004764.s006.docx]

**S6 Table. Country-specific out-of-pocket costs for vaccine-preventable diseases**

| **Country** | **HepB** | **Hib** | **PCV** | **Rota** | **Measles** |
| --- | --- | --- | --- | --- | --- |
| Bangladesh | 382 | 102 | 113 | 73 | 18 |
| Benin | 207 | 53 | 56 | 33 | 10 |
| Burkina Faso | 159 | 43 | 47 | 26 | 8 |
| Burundi | 54 | 30 | 33 | 18 | 8 |
| Cameroon | 139 | 60 | 64 | 35 | 15 |
| Central African Republic | 46 | 28 | 29 | 16 | 9 |
| Chad | 82 | 45 | 50 | 26 | 12 |
| Comoros | 243 | 76 | 82 | 46 | 18 |
| Congo | 75 | 34 | 35 | 20 | 11 |
| Congo, the Democratic Republic of the | 28 | 22 | 23 | 15 | 5 |
| Cote d'Ivoire | 525 | 128 | 135 | 85 | 18 |
| Djibouti | 561 | 159 | 178 | 94 | 36 |
| Ethiopia | 101 | 26 | 29 | 15 | 7 |
| Gambia | 107 | 41 | 43 | 24 | 7 |
| Ghana | 578 | 99 | 105 | 57 | 17 |
| Guinea | 248 | 49 | 53 | 37 | 9 |
| Guinea-Bissau | 164 | 43 | 48 | 25 | 7 |
| Haiti | 227 | 87 | 96 | 60 | 25 |
| India | 518 | 126 | 141 | 92 | 20 |
| Kenya | 147 | 79 | 86 | 48 | 23 |
| Kyrgyzstan | 320 | 66 | 74 | 52 | 13 |
| Lao People's Democratic Republic | 830 | 120 | 133 | 88 | 27 |
| Lesotho | 63 | 68 | 74 | 41 | 14 |
| Liberia | 58 | 40 | 44 | 26 | 11 |
| Madagascar | 80 | 29 | 32 | 18 | 7 |
| Malawi | 21 | 14 | 16 | 4 | 3 |
| Mali | 227 | 55 | 60 | 25 | 9 |
| Mauritania | 302 | 78 | 84 | 48 | 14 |
| Mozambique | 124 | 44 | 49 | 20 | 8 |
| Myanmar | 437 | 82 | 91 | 54 | 10 |
| Nepal | 214 | 62 | 65 | 44 | 11 |
| Nicaragua | 367 | 122 | 129 | 100 | 26 |
| Niger | 102 | 31 | 34 | 20 | 6 |
| Nigeria | 711 | 180 | 192 | 97 | 21 |
| Pakistan | 377 | 103 | 116 | 72 | 17 |
| Papua New Guinea | 290 | 122 | 137 | 86 | 29 |
| Rwanda | 106 | 28 | 33 | 16 | 6 |
| Sao Tome and Principe | 307 | 58 | 62 | 27 | 32 |
| Senegal | 357 | 70 | 74 | 43 | 14 |
| Sierra Leone | 109 | 25 | 27 | 14 | 4 |
| Solomon Islands | 220 | 96 | 100 | 65 | 23 |
| Somalia | 439 | 121 | 133 | 70 | 26 |
| South Sudan | 442 | 129 | 137 | 73 | 29 |
| Sudan | 229 | 59 | 62 | 35 | 11 |
| Tajikistan | 161 | 45 | 51 | 29 | 9 |
| Tanzania, United Republic of | 159 | 47 | 54 | 28 | 10 |
| Togo | 147 | 38 | 40 | 24 | 7 |
| Uganda | 125 | 34 | 38 | 22 | 8 |
| Uzbekistan | 282 | 96 | 108 | 63 | 24 |
| Yemen | 567 | 104 | 112 | 80 | 33 |
| Zambia | 259 | 77 | 87 | 45 | 19 |
| Zimbabwe | 17 | 12 | 13 | 8 | 4 |
