# Supplementary material for: Financial risk protection from vaccines in 52 Gavi-eligible low- and middle-income countries: A modeling study
Source: PLoS Med. 2025 Nov 4;22(11):e1004764. doi: 10.1371/journal.pmed.1004764 (PMC12585062; doi:10.1371/journal.pmed.1004764)
Supplement: S7 Table — (DOCX) [file pmed.1004764.s007.docx]

**S7 Table. Country- and quintile-specific care-seeking rates conditional on disease occurrence**

| **Country** | **Quintile** | **HepB** | **Hib** | **PCV** | **Rota** | **Measles** |
| --- | --- | --- | --- | --- | --- | --- |
| Benin | poorest | 0.39 | 0.39 | 0.12 | 0.39 | 0.26 |
| Benin | poorer | 0.49 | 0.49 | 0.19 | 0.44 | 0.31 |
| Benin | middle | 0.48 | 0.48 | 0.25 | 0.37 | 0.31 |
| Benin | richer | 0.64 | 0.64 | 0.22 | 0.44 | 0.33 |
| Benin | richest | 0.69 | 0.69 | 0.34 | 0.57 | 0.46 |
| Burkina Faso | poorest | 0.67 | 0.67 | 0.24 | 0.56 | 0.40 |
| Burkina Faso | poorer | 0.77 | 0.77 | 0.32 | 0.57 | 0.44 |
| Burkina Faso | middle | 0.73 | 0.73 | 0.28 | 0.60 | 0.44 |
| Burkina Faso | richer | 0.71 | 0.71 | 0.37 | 0.61 | 0.49 |
| Burkina Faso | richest | 0.84 | 0.84 | 0.48 | 0.69 | 0.58 |
| Burundi | poorest | 0.69 | 0.69 | 0.24 | 0.59 | 0.41 |
| Burundi | poorer | 0.70 | 0.70 | 0.25 | 0.55 | 0.40 |
| Burundi | middle | 0.68 | 0.68 | 0.21 | 0.62 | 0.41 |
| Burundi | richer | 0.68 | 0.68 | 0.30 | 0.63 | 0.46 |
| Burundi | richest | 0.74 | 0.74 | 0.37 | 0.66 | 0.51 |
| Cameroon | poorest | 0.47 | 0.47 | 0.24 | 0.49 | 0.36 |
| Cameroon | poorer | 0.61 | 0.61 | 0.30 | 0.50 | 0.40 |
| Cameroon | middle | 0.62 | 0.62 | 0.33 | 0.53 | 0.43 |
| Cameroon | richer | 0.69 | 0.69 | 0.40 | 0.60 | 0.50 |
| Cameroon | richest | 0.71 | 0.71 | 0.43 | 0.48 | 0.45 |
| Congo, the Democratic Republic of the | poorest | 0.43 | 0.43 | 0.30 | 0.44 | 0.37 |
| Congo, the Democratic Republic of the | poorer | 0.54 | 0.54 | 0.38 | 0.47 | 0.43 |
| Congo, the Democratic Republic of the | middle | 0.57 | 0.57 | 0.43 | 0.54 | 0.48 |
| Congo, the Democratic Republic of the | richer | 0.62 | 0.62 | 0.52 | 0.56 | 0.54 |
| Congo, the Democratic Republic of the | richest | 0.70 | 0.70 | 0.55 | 0.57 | 0.56 |
| Congo | poorest | 0.43 | 0.43 | 0.30 | 0.44 | 0.37 |
| Congo | poorer | 0.54 | 0.54 | 0.38 | 0.47 | 0.43 |
| Congo | middle | 0.57 | 0.57 | 0.43 | 0.54 | 0.48 |
| Congo | richer | 0.62 | 0.62 | 0.52 | 0.56 | 0.54 |
| Congo | richest | 0.70 | 0.70 | 0.55 | 0.57 | 0.56 |
| Cote d'Ivoire | poorest | 0.57 | 0.57 | 0.24 | 0.56 | 0.40 |
| Cote d'Ivoire | poorer | 0.61 | 0.61 | 0.32 | 0.57 | 0.44 |
| Cote d'Ivoire | middle | 0.63 | 0.63 | 0.28 | 0.60 | 0.44 |
| Cote d'Ivoire | richer | 0.68 | 0.68 | 0.37 | 0.61 | 0.49 |
| Cote d'Ivoire | richest | 0.75 | 0.75 | 0.48 | 0.69 | 0.58 |
| Ethiopia | poorest | 0.24 | 0.24 | 0.18 | 0.43 | 0.30 |
| Ethiopia | poorer | 0.30 | 0.30 | 0.23 | 0.39 | 0.31 |
| Ethiopia | middle | 0.33 | 0.33 | 0.24 | 0.48 | 0.36 |
| Ethiopia | richer | 0.42 | 0.42 | 0.27 | 0.47 | 0.37 |
| Ethiopia | richest | 0.51 | 0.51 | 0.21 | 0.64 | 0.42 |
| Gambia | poorest | 0.57 | 0.57 | 0.24 | 0.56 | 0.40 |
| Gambia | poorer | 0.61 | 0.61 | 0.32 | 0.57 | 0.44 |
| Gambia | middle | 0.63 | 0.63 | 0.28 | 0.60 | 0.44 |
| Gambia | richer | 0.68 | 0.68 | 0.37 | 0.61 | 0.49 |
| Gambia | richest | 0.75 | 0.75 | 0.48 | 0.69 | 0.58 |
| Ghana | poorest | 0.69 | 0.69 | 0.32 | 0.70 | 0.51 |
| Ghana | poorer | 0.69 | 0.69 | 0.42 | 0.69 | 0.56 |
| Ghana | middle | 0.67 | 0.67 | 0.37 | 0.66 | 0.52 |
| Ghana | richer | 0.65 | 0.65 | 0.49 | 0.75 | 0.62 |
| Ghana | richest | 0.79 | 0.79 | 0.63 | 0.68 | 0.66 |
| Guinea | poorest | 0.39 | 0.39 | 0.28 | 0.50 | 0.39 |
| Guinea | poorer | 0.60 | 0.60 | 0.36 | 0.64 | 0.50 |
| Guinea | middle | 0.66 | 0.66 | 0.32 | 0.71 | 0.51 |
| Guinea | richer | 0.71 | 0.71 | 0.42 | 0.75 | 0.59 |
| Guinea | richest | 0.79 | 0.79 | 0.55 | 0.78 | 0.67 |
| Haiti | poorest | 0.31 | 0.31 | 0.21 | 0.30 | 0.26 |
| Haiti | poorer | 0.47 | 0.47 | 0.20 | 0.35 | 0.28 |
| Haiti | middle | 0.52 | 0.52 | 0.32 | 0.40 | 0.36 |
| Haiti | richer | 0.49 | 0.49 | 0.38 | 0.43 | 0.41 |
| Haiti | richest | 0.66 | 0.66 | 0.38 | 0.46 | 0.42 |
| Kenya | poorest | 0.63 | 0.63 | 0.52 | 0.68 | 0.60 |
| Kenya | poorer | 0.73 | 0.73 | 0.48 | 0.64 | 0.56 |
| Kenya | middle | 0.74 | 0.74 | 0.55 | 0.69 | 0.62 |
| Kenya | richer | 0.78 | 0.78 | 0.65 | 0.65 | 0.65 |
| Kenya | richest | 0.77 | 0.77 | 0.61 | 0.62 | 0.62 |
| Lao People's Democratic Republic | poorest | 0.52 | 0.52 | 0.49 | 0.63 | 0.56 |
| Lao People's Democratic Republic | poorer | 0.56 | 0.56 | 0.49 | 0.67 | 0.58 |
| Lao People's Democratic Republic | middle | 0.57 | 0.57 | 0.53 | 0.65 | 0.59 |
| Lao People's Democratic Republic | richer | 0.68 | 0.68 | 0.38 | 0.68 | 0.53 |
| Lao People's Democratic Republic | richest | 0.71 | 0.71 | 0.56 | 0.71 | 0.64 |
| Malawi | poorest | 0.59 | 0.59 | 0.45 | 0.68 | 0.57 |
| Malawi | poorer | 0.47 | 0.47 | 0.37 | 0.63 | 0.50 |
| Malawi | middle | 0.57 | 0.57 | 0.46 | 0.68 | 0.57 |
| Malawi | richer | 0.53 | 0.53 | 0.42 | 0.70 | 0.56 |
| Malawi | richest | 0.57 | 0.57 | 0.47 | 0.64 | 0.56 |
| Mali | poorest | 0.49 | 0.49 | 0.25 | 0.47 | 0.36 |
| Mali | poorer | 0.44 | 0.44 | 0.20 | 0.45 | 0.32 |
| Mali | middle | 0.49 | 0.49 | 0.07 | 0.48 | 0.28 |
| Mali | richer | 0.59 | 0.59 | 0.40 | 0.51 | 0.45 |
| Mali | richest | 0.72 | 0.72 | 0.24 | 0.58 | 0.41 |
| Mozambique | poorest | 0.58 | 0.58 | 0.50 | 0.45 | 0.48 |
| Mozambique | poorer | 0.67 | 0.67 | 0.49 | 0.51 | 0.50 |
| Mozambique | middle | 0.71 | 0.71 | 0.50 | 0.72 | 0.61 |
| Mozambique | richer | 0.78 | 0.78 | 0.74 | 0.74 | 0.74 |
| Mozambique | richest | 0.83 | 0.83 | 0.65 | 0.62 | 0.63 |
| Myanmar | poorest | 0.63 | 0.63 | 0.48 | 0.61 | 0.55 |
| Myanmar | poorer | 0.66 | 0.66 | 0.48 | 0.64 | 0.56 |
| Myanmar | middle | 0.58 | 0.58 | 0.52 | 0.62 | 0.57 |
| Myanmar | richer | 0.78 | 0.78 | 0.37 | 0.76 | 0.56 |
| Myanmar | richest | 0.77 | 0.77 | 0.55 | 0.82 | 0.68 |
| Nepal | poorest | 0.72 | 0.72 | 0.34 | 0.55 | 0.44 |
| Nepal | poorer | 0.75 | 0.75 | 0.52 | 0.67 | 0.60 |
| Nepal | middle | 0.75 | 0.75 | 0.33 | 0.85 | 0.59 |
| Nepal | richer | 0.77 | 0.77 | 0.50 | 0.69 | 0.59 |
| Nepal | richest | 0.80 | 0.80 | 0.37 | 0.72 | 0.55 |
| Niger | poorest | 0.51 | 0.51 | 0.06 | 0.48 | 0.27 |
| Niger | poorer | 0.58 | 0.58 | 0.05 | 0.56 | 0.30 |
| Niger | middle | 0.64 | 0.64 | 0.02 | 0.68 | 0.35 |
| Niger | richer | 0.69 | 0.69 | 0.06 | 0.66 | 0.36 |
| Niger | richest | 0.71 | 0.71 | 0.32 | 0.62 | 0.47 |
| Nigeria | poorest | 0.68 | 0.68 | 0.27 | 0.60 | 0.44 |
| Nigeria | poorer | 0.70 | 0.70 | 0.25 | 0.63 | 0.44 |
| Nigeria | middle | 0.72 | 0.72 | 0.31 | 0.66 | 0.49 |
| Nigeria | richer | 0.79 | 0.79 | 0.22 | 0.65 | 0.44 |
| Nigeria | richest | 0.85 | 0.85 | 0.34 | 0.81 | 0.58 |
| Sao Tome and Principe | poorest | 0.43 | 0.43 | 0.30 | 0.44 | 0.37 |
| Sao Tome and Principe | poorer | 0.54 | 0.54 | 0.38 | 0.47 | 0.43 |
| Sao Tome and Principe | middle | 0.57 | 0.57 | 0.43 | 0.54 | 0.48 |
| Sao Tome and Principe | richer | 0.62 | 0.62 | 0.52 | 0.56 | 0.54 |
| Sao Tome and Principe | richest | 0.70 | 0.70 | 0.55 | 0.57 | 0.56 |
| Senegal | poorest | 0.46 | 0.46 | 0.18 | 0.44 | 0.31 |
| Senegal | poorer | 0.44 | 0.44 | 0.47 | 0.42 | 0.45 |
| Senegal | middle | 0.48 | 0.48 | 0.39 | 0.46 | 0.43 |
| Senegal | richer | 0.55 | 0.55 | 0.50 | 0.46 | 0.48 |
| Senegal | richest | 0.58 | 0.58 | 0.09 | 0.59 | 0.34 |
| Sierra Leone | poorest | 0.77 | 0.77 | 0.31 | 0.77 | 0.54 |
| Sierra Leone | poorer | 0.75 | 0.75 | 0.40 | 0.73 | 0.57 |
| Sierra Leone | middle | 0.76 | 0.76 | 0.36 | 0.79 | 0.58 |
| Sierra Leone | richer | 0.73 | 0.73 | 0.47 | 0.72 | 0.59 |
| Sierra Leone | richest | 0.77 | 0.77 | 0.61 | 0.72 | 0.66 |
| Tajikistan | poorest | 0.36 | 0.36 | 0.66 | 0.49 | 0.57 |
| Tajikistan | poorer | 0.46 | 0.46 | 0.85 | 0.51 | 0.68 |
| Tajikistan | middle | 0.37 | 0.37 | 0.71 | 0.54 | 0.62 |
| Tajikistan | richer | 0.58 | 0.58 | 0.78 | 0.59 | 0.69 |
| Tajikistan | richest | 0.49 | 0.49 | 0.77 | 0.48 | 0.63 |
| Tanzania, United Republic of | poorest | 0.68 | 0.68 | 0.61 | 0.65 | 0.63 |
| Tanzania, United Republic of | poorer | 0.77 | 0.77 | 0.50 | 0.73 | 0.62 |
| Tanzania, United Republic of | middle | 0.75 | 0.75 | 0.66 | 0.72 | 0.69 |
| Tanzania, United Republic of | richer | 0.82 | 0.82 | 0.70 | 0.70 | 0.70 |
| Tanzania, United Republic of | richest | 0.76 | 0.76 | 0.67 | 0.73 | 0.70 |
| Uganda | poorest | 0.85 | 0.85 | 0.37 | 0.73 | 0.55 |
| Uganda | poorer | 0.86 | 0.86 | 0.46 | 0.69 | 0.57 |
| Uganda | middle | 0.89 | 0.89 | 0.45 | 0.68 | 0.57 |
| Uganda | richer | 0.86 | 0.86 | 0.43 | 0.66 | 0.55 |
| Uganda | richest | 0.92 | 0.92 | 0.63 | 0.69 | 0.66 |
| Zimbabwe | poorest | 0.49 | 0.49 | 0.35 | 0.40 | 0.37 |
| Zimbabwe | poorer | 0.45 | 0.45 | 0.38 | 0.43 | 0.40 |
| Zimbabwe | middle | 0.45 | 0.45 | 0.37 | 0.40 | 0.39 |
| Zimbabwe | richer | 0.51 | 0.51 | 0.32 | 0.40 | 0.36 |
| Zimbabwe | richest | 0.64 | 0.64 | 0.66 | 0.53 | 0.60 |
| Bangladesh | poorest | 0.57 | 0.57 | 0.50 | 0.59 | 0.55 |
| Bangladesh | poorer | 0.60 | 0.60 | 0.55 | 0.63 | 0.59 |
| Bangladesh | middle | 0.59 | 0.59 | 0.53 | 0.63 | 0.58 |
| Bangladesh | richer | 0.68 | 0.68 | 0.49 | 0.66 | 0.58 |
| Bangladesh | richest | 0.71 | 0.71 | 0.56 | 0.66 | 0.61 |
| Central African Republic | poorest | 0.55 | 0.55 | 0.30 | 0.54 | 0.42 |
| Central African Republic | poorer | 0.60 | 0.60 | 0.34 | 0.55 | 0.45 |
| Central African Republic | middle | 0.62 | 0.62 | 0.35 | 0.60 | 0.47 |
| Central African Republic | richer | 0.67 | 0.67 | 0.42 | 0.61 | 0.51 |
| Central African Republic | richest | 0.73 | 0.73 | 0.47 | 0.64 | 0.56 |
| Chad | poorest | 0.55 | 0.55 | 0.30 | 0.54 | 0.42 |
| Chad | poorer | 0.60 | 0.60 | 0.34 | 0.55 | 0.45 |
| Chad | middle | 0.62 | 0.62 | 0.35 | 0.60 | 0.47 |
| Chad | richer | 0.67 | 0.67 | 0.42 | 0.61 | 0.51 |
| Chad | richest | 0.73 | 0.73 | 0.47 | 0.64 | 0.56 |
| Comoros | poorest | 0.55 | 0.55 | 0.30 | 0.54 | 0.42 |
| Comoros | poorer | 0.60 | 0.60 | 0.34 | 0.55 | 0.45 |
| Comoros | middle | 0.62 | 0.62 | 0.35 | 0.60 | 0.47 |
| Comoros | richer | 0.67 | 0.67 | 0.42 | 0.61 | 0.51 |
| Comoros | richest | 0.73 | 0.73 | 0.47 | 0.64 | 0.56 |
| Djibouti | poorest | 0.55 | 0.55 | 0.30 | 0.54 | 0.42 |
| Djibouti | poorer | 0.60 | 0.60 | 0.34 | 0.55 | 0.45 |
| Djibouti | middle | 0.62 | 0.62 | 0.35 | 0.60 | 0.47 |
| Djibouti | richer | 0.67 | 0.67 | 0.42 | 0.61 | 0.51 |
| Djibouti | richest | 0.73 | 0.73 | 0.47 | 0.64 | 0.56 |
| Guinea-Bissau | poorest | 0.55 | 0.55 | 0.30 | 0.54 | 0.42 |
| Guinea-Bissau | poorer | 0.60 | 0.60 | 0.34 | 0.55 | 0.45 |
| Guinea-Bissau | middle | 0.62 | 0.62 | 0.35 | 0.60 | 0.47 |
| Guinea-Bissau | richer | 0.67 | 0.67 | 0.42 | 0.61 | 0.51 |
| Guinea-Bissau | richest | 0.73 | 0.73 | 0.47 | 0.64 | 0.56 |
| India | poorest | 0.57 | 0.57 | 0.50 | 0.59 | 0.55 |
| India | poorer | 0.60 | 0.60 | 0.55 | 0.63 | 0.59 |
| India | middle | 0.59 | 0.59 | 0.53 | 0.63 | 0.58 |
| India | richer | 0.68 | 0.68 | 0.49 | 0.66 | 0.58 |
| India | richest | 0.71 | 0.71 | 0.56 | 0.66 | 0.61 |
| Kyrgyzstan | poorest | 0.57 | 0.57 | 0.50 | 0.59 | 0.55 |
| Kyrgyzstan | poorer | 0.60 | 0.60 | 0.55 | 0.63 | 0.59 |
| Kyrgyzstan | middle | 0.59 | 0.59 | 0.53 | 0.63 | 0.58 |
| Kyrgyzstan | richer | 0.68 | 0.68 | 0.49 | 0.66 | 0.58 |
| Kyrgyzstan | richest | 0.71 | 0.71 | 0.56 | 0.66 | 0.61 |
| Lesotho | poorest | 0.55 | 0.55 | 0.30 | 0.54 | 0.42 |
| Lesotho | poorer | 0.60 | 0.60 | 0.34 | 0.55 | 0.45 |
| Lesotho | middle | 0.62 | 0.62 | 0.35 | 0.60 | 0.47 |
| Lesotho | richer | 0.67 | 0.67 | 0.42 | 0.61 | 0.51 |
| Lesotho | richest | 0.73 | 0.73 | 0.47 | 0.64 | 0.56 |
| Liberia | poorest | 0.55 | 0.55 | 0.30 | 0.54 | 0.42 |
| Liberia | poorer | 0.60 | 0.60 | 0.34 | 0.55 | 0.45 |
| Liberia | middle | 0.62 | 0.62 | 0.35 | 0.60 | 0.47 |
| Liberia | richer | 0.67 | 0.67 | 0.42 | 0.61 | 0.51 |
| Liberia | richest | 0.73 | 0.73 | 0.47 | 0.64 | 0.56 |
| Madagascar | poorest | 0.55 | 0.55 | 0.30 | 0.54 | 0.42 |
| Madagascar | poorer | 0.60 | 0.60 | 0.34 | 0.55 | 0.45 |
| Madagascar | middle | 0.62 | 0.62 | 0.35 | 0.60 | 0.47 |
| Madagascar | richer | 0.67 | 0.67 | 0.42 | 0.61 | 0.51 |
| Madagascar | richest | 0.73 | 0.73 | 0.47 | 0.64 | 0.56 |
| Mauritania | poorest | 0.55 | 0.55 | 0.30 | 0.54 | 0.42 |
| Mauritania | poorer | 0.60 | 0.60 | 0.34 | 0.55 | 0.45 |
| Mauritania | middle | 0.62 | 0.62 | 0.35 | 0.60 | 0.47 |
| Mauritania | richer | 0.67 | 0.67 | 0.42 | 0.61 | 0.51 |
| Mauritania | richest | 0.73 | 0.73 | 0.47 | 0.64 | 0.56 |
| Nicaragua | poorest | 0.31 | 0.31 | 0.21 | 0.30 | 0.26 |
| Nicaragua | poorer | 0.47 | 0.47 | 0.20 | 0.35 | 0.28 |
| Nicaragua | middle | 0.52 | 0.52 | 0.32 | 0.40 | 0.36 |
| Nicaragua | richer | 0.49 | 0.49 | 0.38 | 0.43 | 0.41 |
| Nicaragua | richest | 0.66 | 0.66 | 0.38 | 0.46 | 0.42 |
| Pakistan | poorest | 0.57 | 0.57 | 0.50 | 0.59 | 0.55 |
| Pakistan | poorer | 0.60 | 0.60 | 0.55 | 0.63 | 0.59 |
| Pakistan | middle | 0.59 | 0.59 | 0.53 | 0.63 | 0.58 |
| Pakistan | richer | 0.68 | 0.68 | 0.49 | 0.66 | 0.58 |
| Pakistan | richest | 0.71 | 0.71 | 0.56 | 0.66 | 0.61 |
| Papua New Guinea | poorest | 0.31 | 0.31 | 0.17 | 0.35 | 0.26 |
| Papua New Guinea | poorer | 0.44 | 0.44 | 0.20 | 0.30 | 0.25 |
| Papua New Guinea | middle | 0.48 | 0.48 | 0.41 | 0.30 | 0.35 |
| Papua New Guinea | richer | 0.62 | 0.62 | 0.29 | 0.41 | 0.35 |
| Papua New Guinea | richest | 0.66 | 0.66 | 0.48 | 0.51 | 0.50 |
| Rwanda | poorest | 0.55 | 0.55 | 0.30 | 0.54 | 0.42 |
| Rwanda | poorer | 0.60 | 0.60 | 0.34 | 0.55 | 0.45 |
| Rwanda | middle | 0.62 | 0.62 | 0.35 | 0.60 | 0.47 |
| Rwanda | richer | 0.67 | 0.67 | 0.42 | 0.61 | 0.51 |
| Rwanda | richest | 0.73 | 0.73 | 0.47 | 0.64 | 0.56 |
| Solomon Islands | poorest | 0.31 | 0.31 | 0.17 | 0.35 | 0.26 |
| Solomon Islands | poorer | 0.44 | 0.44 | 0.20 | 0.30 | 0.25 |
| Solomon Islands | middle | 0.48 | 0.48 | 0.41 | 0.30 | 0.35 |
| Solomon Islands | richer | 0.62 | 0.62 | 0.29 | 0.41 | 0.35 |
| Solomon Islands | richest | 0.66 | 0.66 | 0.48 | 0.51 | 0.50 |
| Somalia | poorest | 0.55 | 0.55 | 0.30 | 0.54 | 0.42 |
| Somalia | poorer | 0.60 | 0.60 | 0.34 | 0.55 | 0.45 |
| Somalia | middle | 0.62 | 0.62 | 0.35 | 0.60 | 0.47 |
| Somalia | richer | 0.67 | 0.67 | 0.42 | 0.61 | 0.51 |
| Somalia | richest | 0.73 | 0.73 | 0.47 | 0.64 | 0.56 |
| South Sudan | poorest | 0.55 | 0.55 | 0.30 | 0.54 | 0.42 |
| South Sudan | poorer | 0.60 | 0.60 | 0.34 | 0.55 | 0.45 |
| South Sudan | middle | 0.62 | 0.62 | 0.35 | 0.60 | 0.47 |
| South Sudan | richer | 0.67 | 0.67 | 0.42 | 0.61 | 0.51 |
| South Sudan | richest | 0.73 | 0.73 | 0.47 | 0.64 | 0.56 |
| Sudan | poorest | 0.55 | 0.55 | 0.30 | 0.54 | 0.42 |
| Sudan | poorer | 0.60 | 0.60 | 0.34 | 0.55 | 0.45 |
| Sudan | middle | 0.62 | 0.62 | 0.35 | 0.60 | 0.47 |
| Sudan | richer | 0.67 | 0.67 | 0.42 | 0.61 | 0.51 |
| Sudan | richest | 0.73 | 0.73 | 0.47 | 0.64 | 0.56 |
| Togo | poorest | 0.55 | 0.55 | 0.30 | 0.54 | 0.42 |
| Togo | poorer | 0.60 | 0.60 | 0.34 | 0.55 | 0.45 |
| Togo | middle | 0.62 | 0.62 | 0.35 | 0.60 | 0.47 |
| Togo | richer | 0.67 | 0.67 | 0.42 | 0.61 | 0.51 |
| Togo | richest | 0.73 | 0.73 | 0.47 | 0.64 | 0.56 |
| Uzbekistan | poorest | 0.57 | 0.57 | 0.50 | 0.59 | 0.55 |
| Uzbekistan | poorer | 0.60 | 0.60 | 0.55 | 0.63 | 0.59 |
| Uzbekistan | middle | 0.59 | 0.59 | 0.53 | 0.63 | 0.58 |
| Uzbekistan | richer | 0.68 | 0.68 | 0.49 | 0.66 | 0.58 |
| Uzbekistan | richest | 0.71 | 0.71 | 0.56 | 0.66 | 0.61 |
| Yemen | poorest | 0.57 | 0.57 | 0.50 | 0.59 | 0.55 |
| Yemen | poorer | 0.60 | 0.60 | 0.55 | 0.63 | 0.59 |
| Yemen | middle | 0.59 | 0.59 | 0.53 | 0.63 | 0.58 |
| Yemen | richer | 0.68 | 0.68 | 0.49 | 0.66 | 0.58 |
| Yemen | richest | 0.71 | 0.71 | 0.56 | 0.66 | 0.61 |
| Zambia | poorest | 0.55 | 0.55 | 0.30 | 0.54 | 0.42 |
| Zambia | poorer | 0.60 | 0.60 | 0.34 | 0.55 | 0.45 |
| Zambia | middle | 0.62 | 0.62 | 0.35 | 0.60 | 0.47 |
| Zambia | richer | 0.67 | 0.67 | 0.42 | 0.61 | 0.51 |
| Zambia | richest | 0.73 | 0.73 | 0.47 | 0.64 | 0.56 |
