# Supplementary material for: Financial risk protection from vaccines in 52 Gavi-eligible low- and middle-income countries: A modeling study
Source: PLoS Med. 2025 Nov 4;22(11):e1004764. doi: 10.1371/journal.pmed.1004764 (PMC12585062; doi:10.1371/journal.pmed.1004764)
Supplement: S8 Table — (DOCX) [file pmed.1004764.s008.docx]

**S8 Table.** Vaccine impact on cases of catastrophic health expenditures (CHE) and impoverishing health expenditure (IHE) averted per 10,000 and total cases of CHE, from 2000-to-2030 vaccinee cohorts.

| **Vaccine** | **Quintile** | **Total CHE cases averted (10%, thousands)** | **CHE cases averted per 10,000 (10%)** | **Total CHE cases averted (25%, thousands)** | **CHE cases averted per 10,000 (25%)** | **Total CHE cases averted (40%, thousands)** | **CHE cases averted per 10,000 (40%)** | **Total IHE cases averted (thousands)** | **IHE cases averted per 10,000** |
| --- | --- | --- | --- | --- | --- | --- | --- | --- | --- |
| HepB | Poorest | 11950 | 442 | 11845 | 438 | 11534 | 427 | 12000 | 444 |
| HepB | Poorer | 13566 | 502 | 12820 | 474 | 11919 | 441 | 13668 | 506 |
| HepB | Middle | 12324 | 456 | 10921 | 404 | 8294 | 307 | 12781 | 473 |
| HepB | Richer | 13981 | 517 | 10515 | 389 | 6057 | 224 | 11353 | 420 |
| HepB | Richest | 12608 | 467 | 6016 | 223 | 1462 | 54 | 4692 | 174 |
| HepB BD | Poorest | 249 | 34 | 249 | 34 | 249 | 34 | 249 | 34 |
| HepB BD | Poorer | 258 | 35 | 258 | 35 | 253 | 35 | 258 | 35 |
| HepB BD | Middle | 299 | 41 | 298 | 41 | 283 | 39 | 299 | 41 |
| HepB BD | Richer | 387 | 53 | 349 | 48 | 257 | 35 | 198 | 27 |
| HepB BD | Richest | 331 | 45 | 181 | 25 | 54 | 7 | 53 | 7 |
| Hib3 | Poorest | 3141 | 136 | 2784 | 121 | 1706 | 74 | 3161 | 137 |
| Hib3 | Poorer | 3910 | 169 | 835 | 36 | 61 | 3 | 3922 | 170 |
| Hib3 | Middle | 2945 | 128 | 36 | 2 | 5 | 0 | 3180 | 138 |
| Hib3 | Richer | 2282 | 99 | 5 | 0 | 0 | 0 | 1552 | 67 |
| Hib3 | Richest | 280 | 12 | 0 | 0 | 0 | 0 | 539 | 23 |
| PCV3 | Poorest | 8688 | 526 | 8230 | 498 | 5721 | 346 | 8721 | 528 |
| PCV3 | Poorer | 9467 | 573 | 4011 | 243 | 145 | 9 | 9594 | 581 |
| PCV3 | Middle | 7898 | 478 | 76 | 5 | 16 | 1 | 8278 | 501 |
| PCV3 | Richer | 4262 | 258 | 19 | 1 | 0 | 0 | 3540 | 214 |
| PCV3 | Richest | 1749 | 106 | 0 | 0 | 0 | 0 | 1459 | 88 |
| Rota | Poorest | 16313 | 1084 | 10673 | 709 | 5159 | 343 | 16960 | 1127 |
| Rota | Poorer | 13506 | 898 | 102 | 7 | 6 | 0 | 17028 | 1132 |
| Rota | Middle | 8008 | 532 | 6 | 0 | 0 | 0 | 15993 | 1063 |
| Rota | Richer | 1626 | 108 | 0 | 0 | 0 | 0 | 6497 | 432 |
| Rota | Richest | 3 | 0 | 0 | 0 | 0 | 0 | 2715 | 180 |
| MCV1 | Poorest | 44229 | 1381 | 9898 | 309 | 4929 | 154 | 136829 | 4272 |
| MCV1 | Poorer | 1135 | 35 | 3 | 0 | 0 | 0 | 143797 | 4490 |
| MCV1 | Middle | 142 | 4 | 0 | 0 | 0 | 0 | 119670 | 3736 |
| MCV1 | Richer | 0 | 0 | 0 | 0 | 0 | 0 | 42286 | 1320 |
| MCV1 | Richest | 0 | 0 | 0 | 0 | 0 | 0 | 17776 | 555 |
| MCV2 | Poorest | 5540 | 342 | 1053 | 65 | 499 | 31 | 15212 | 938 |
| MCV2 | Poorer | 70 | 4 | 0 | 0 | 0 | 0 | 15936 | 983 |
| MCV2 | Middle | 0 | 0 | 0 | 0 | 0 | 0 | 12954 | 799 |
| MCV2 | Richer | 0 | 0 | 0 | 0 | 0 | 0 | 3713 | 229 |
| MCV2 | Richest | 0 | 0 | 0 | 0 | 0 | 0 | 1727 | 107 |
| SIA | Poorest | 7412 | 157 | 1844 | 39 | 897 | 19 | 24371 | 516 |
| SIA | Poorer | 341 | 7 | 0 | 0 | 0 | 0 | 22619 | 479 |
| SIA | Middle | 55 | 1 | 0 | 0 | 0 | 0 | 18442 | 390 |
| SIA | Richer | 0 | 0 | 0 | 0 | 0 | 0 | 13648 | 289 |
| SIA | Richest | 0 | 0 | 0 | 0 | 0 | 0 | 6983 | 148 |

HepB: routine three infant doses of hepatitis B vaccine; HepB BD: birth dose of hepatitis B vaccine given alone; Hib3: routine three infant doses of *Haemophilus influenzae* type B vaccine; PCV3: routine three doses of *Streptococcus pneumoniae* vaccine; Rota: routine two infant doses of rotavirus vaccine; MCV1: routine first dose of measles vaccine; MCV2: routine second dose of measles vaccine; SIA: campaign measles vaccine.
