# Supplementary material for: Financial risk protection from vaccines in 52 Gavi-eligible low- and middle-income countries: A modeling study
Source: PLoS Med. 2025 Nov 4;22(11):e1004764. doi: 10.1371/journal.pmed.1004764 (PMC12585062; doi:10.1371/journal.pmed.1004764)
Supplement: S1 Fig — (DOCX) [file pmed.1004764.s009.docx]

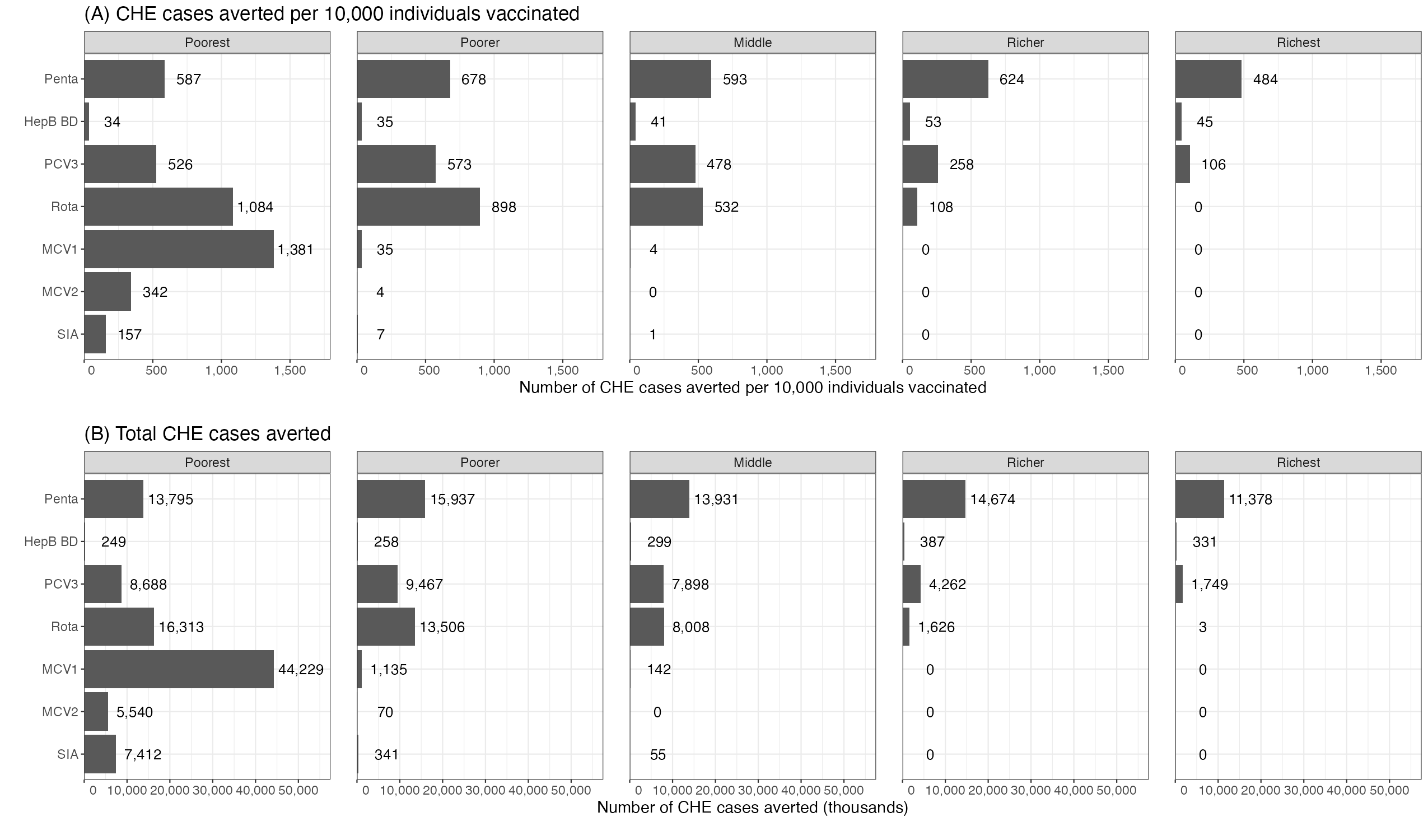


**S1 Fig.** Vaccine impact including pentavalent vaccine (HepB and Hib3) on cases of catastrophic health expenditures (CHE) averted per 10,000 (A) and total cases of CHE (B), from 2000-to-2030 vaccinee cohorts, at a 10% CHE threshold of consumption.

Penta: pentavalent vaccine for prevention of hepatitis B and *Haemophilus influenzae* type; HepB BD: birth dose of hepatitis B vaccine given alone; PCV3: routine three doses of *Streptococcus pneumoniae* vaccine; Rota: routine two infant doses of rotavirus vaccine; MCV1: routine first dose of measles vaccine; MCV2: routine second dose of measles vaccine; SIA: campaign measles vaccine.
