# Supplementary material for: Financial risk protection from vaccines in 52 Gavi-eligible low- and middle-income countries: A modeling study
Source: PLoS Med. 2025 Nov 4;22(11):e1004764. doi: 10.1371/journal.pmed.1004764 (PMC12585062; doi:10.1371/journal.pmed.1004764)
Supplement: S6 Fig — (DOCX) [file pmed.1004764.s014.docx]

**
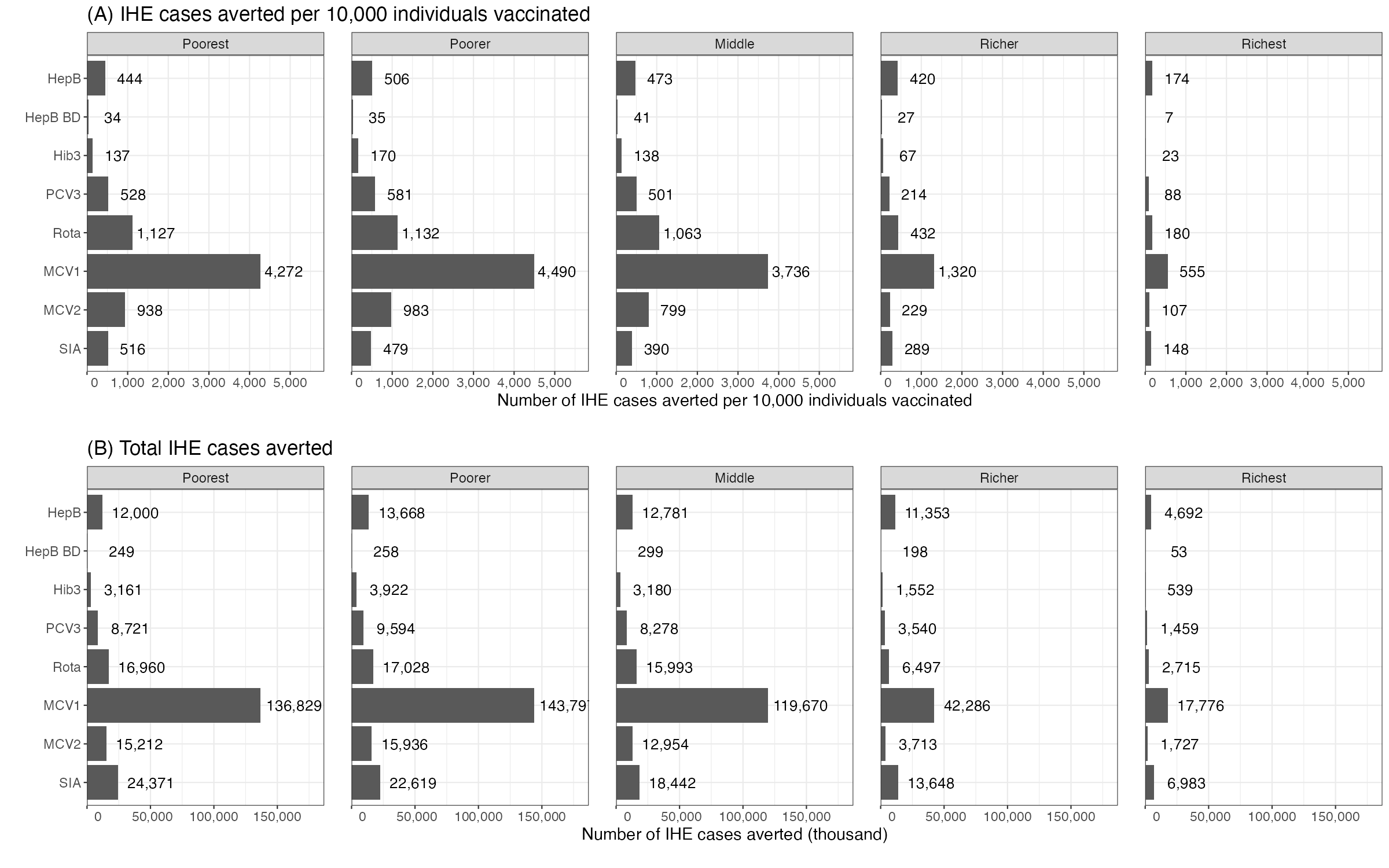
**

**S6 Fig.** Vaccine impact on cases of impoverishing health expenditures (IHE) averted among 2000–2030 vaccinee cohorts, using a $2.15-per-day poverty threshold.

HepB: routine three infant doses of hepatitis B vaccine; HepB BD: birth dose of hepatitis B vaccine given alone; Hib3: routine three infant doses of *Haemophilus influenzae* type B vaccine; PCV3: routine three doses of *Streptococcus pneumoniae* vaccine; Rota: routine two infant doses of rotavirus vaccine; MCV1: routine first dose of measles vaccine; MCV2: routine second dose of measles vaccine; SIA: campaign measles vaccine.,
