# Supplementary material for: Financial risk protection from vaccines in 52 Gavi-eligible low- and middle-income countries: A modeling study
Source: PLoS Med. 2025 Nov 4;22(11):e1004764. doi: 10.1371/journal.pmed.1004764 (PMC12585062; doi:10.1371/journal.pmed.1004764)
Supplement: S8 Fig — (DOCX) [file pmed.1004764.s016.docx]

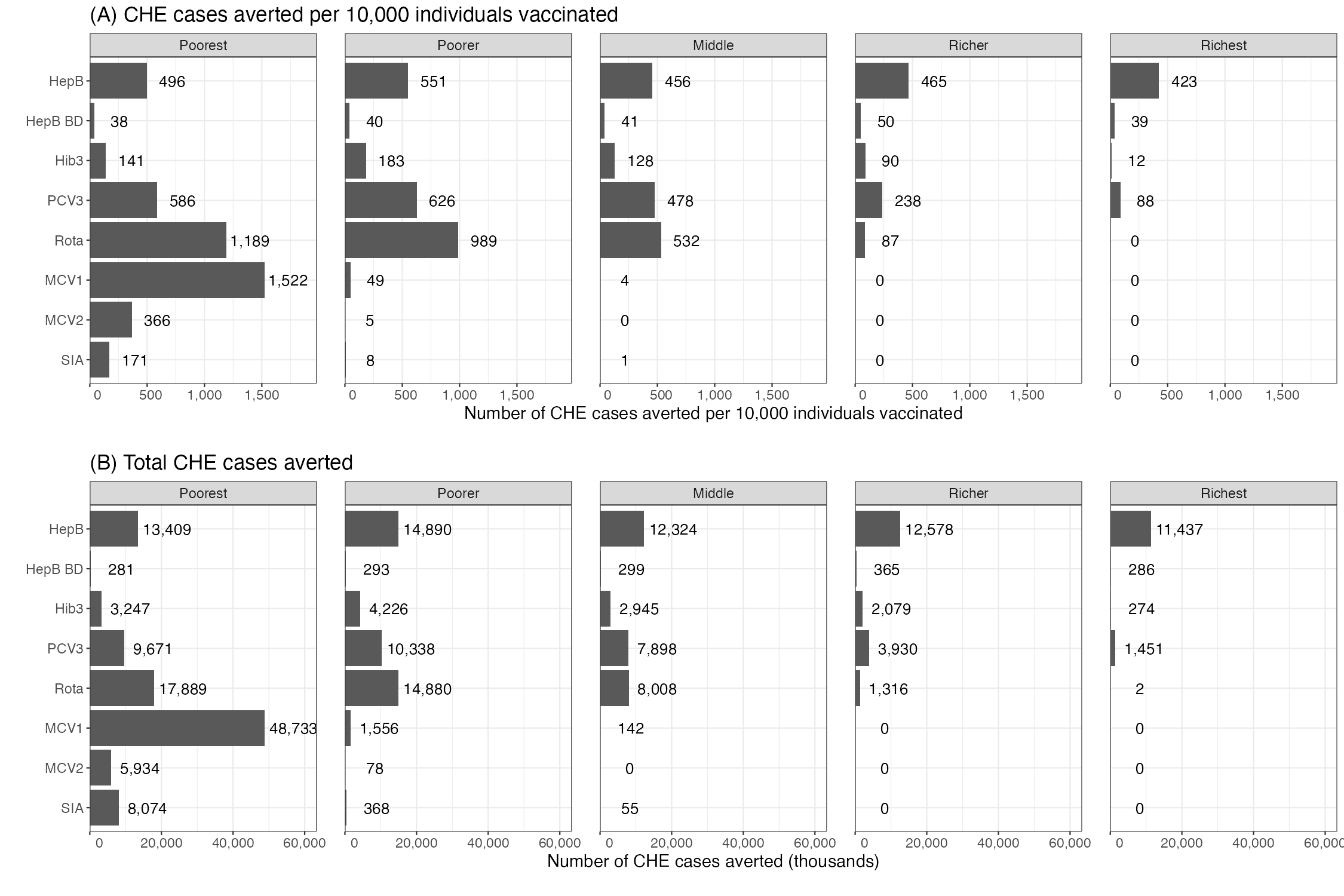


**S8 Fig.** Sensitivity analysis results: adjusted disease probability without immunization by wealth quintile – a 10% increase for the poorest and poorer, and a 10% decrease for the richest and richer, at a 10% catastrophic health expenditures (CHE) threshold of consumption.

HepB: routine three infant doses of hepatitis B vaccine; HepB BD: birth dose of hepatitis B vaccine given alone; Hib3: routine three infant doses of *Haemophilus influenzae* type B vaccine; PCV3: routine three doses of *Streptococcus pneumoniae* vaccine; Rota: routine two infant doses of rotavirus vaccine; MCV1: routine first dose of measles vaccine; MCV2: routine second dose of measles vaccine; SIA: campaign measles vaccine.
