# Supplementary material for: Financial risk protection from vaccines in 52 Gavi-eligible low- and middle-income countries: A modeling study
Source: PLoS Med. 2025 Nov 4;22(11):e1004764. doi: 10.1371/journal.pmed.1004764 (PMC12585062; doi:10.1371/journal.pmed.1004764)
Supplement: S11 Fig — (DOCX) [file pmed.1004764.s019.docx]

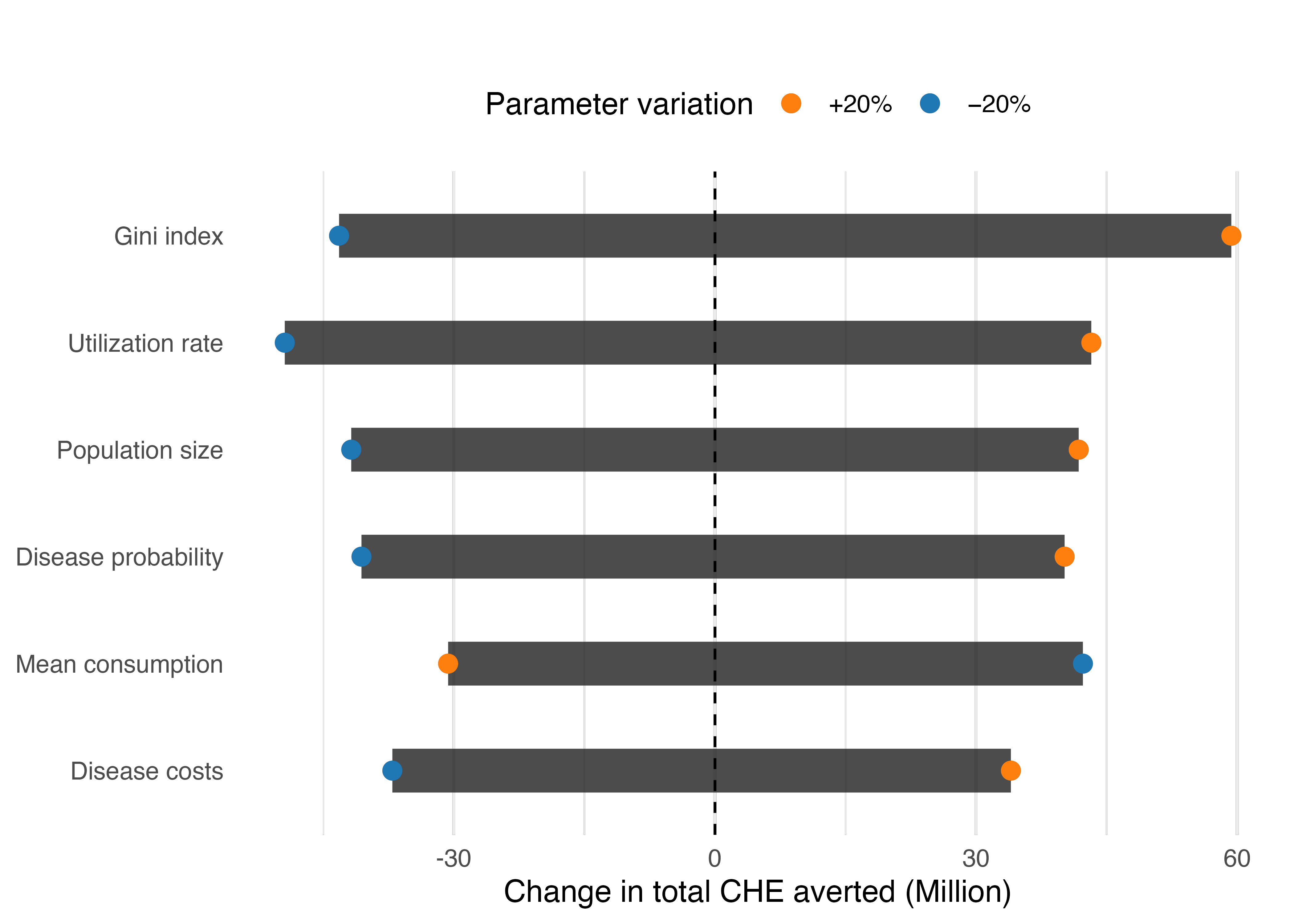


**S11 Fig.** One-way sensitivity analysis of key parameters on total catastrophic health expenditures (CHE) averted
