# Supplementary material for: Financial risk protection from vaccines in 52 Gavi-eligible low- and middle-income countries: A modeling study
Source: PLoS Med. 2025 Nov 4;22(11):e1004764. doi: 10.1371/journal.pmed.1004764 (PMC12585062; doi:10.1371/journal.pmed.1004764)
Supplement: S12 Fig — (DOCX) [file pmed.1004764.s020.docx]

**
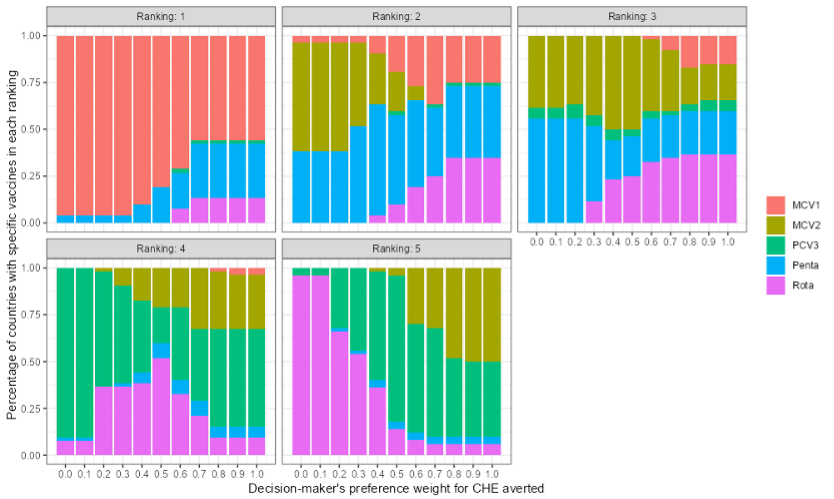
**

**S12 Fig.** Percentage of countries with specific vaccines in each aggregated ranking over a range of preference weights (from 0 to 1) for catastrophic health expenditures (CHE).

PCV3: routine three doses of *Streptococcus pneumoniae* vaccine; Rota: routine two infant doses of rotavirus vaccine; MCV1: routine first dose of measles vaccine; MCV2: routine second dose of measles vaccine; SIA: campaign measles vaccine; Penta: pentavalent vaccine for prevention of hepatitis B and *Haemophilus influenzae* type.
